# Supplementary material for: Mapping of Ebola virus spillover: Suitability and seasonal variability at the landscape scale
Source: PLoS Negl Trop Dis. 2021 Aug 23;15(8):e0009683. doi: 10.1371/journal.pntd.0009683 (PMC8425568; doi:10.1371/journal.pntd.0009683)
Supplement: S1 Text — (DOCX) [file pntd.0009683.s002.docx]

**S1 Text.**  **Sources of data, spatial data manipulation and estimation of variables associated with risk factors of EBOV spillover**

All spatial data used in this study came from open access databases (Table A). Risk factors, their associated variable and the spatial and temporal resolutions of the collected data layers are shown in Table B.

All spatial data were transformed into a raster layer if it were in a different format. All data raster layers need to be of the same spatial resolution to be integrated in a GIS-MCE. Here we used a resolution of 1 km x 1 km as we considered it appropriate for a study at a regional scale (The exact resolution was 918.57 x 921.39 m for Guinée forestière, 927.44 x 921.39 m for the area in Congo, and 927.64 x 921.5 m for the area in Gabon). Local projections, namely Dabola 1981, UTM 33S and Gabon TM, were used for Guinée forestière, the Congo area and the Gabon area, respectively. Table C shows the GIS manipulations to obtain each of the risk factor raster layers.

Once all risk factor raster layers were in the same resolution they were standardized to a continuous scale so that all would have pixel values ranging from 0 to 1 (i.e. low to high suitability) using the following equation:

$X^{S}i,j=\frac{\left( X_{i,j}-{min}_{j} \right)}{\left( {max}_{j}-{min}_{j} \right)}$

Where *X_i_,_j_* is the value of risk factor *j* in pixel *i,* *X^S^_i,j_* is the standardized value and *min_j_* and *max_j_* are minimal and maximal values of risk factor *j*. Then we used fuzzy membership functions to describe the relationship between each risk factor and the outcome (i.e. possibility of EBOV spillover) (Table D and Fig A).

Location of villages in the areas of study was obtained from OpenStreetMap with the key ‘place’. We then filtered to keep those places considered a ‘village’, which refers to places with more inhabitants than a ‘hamlet’, but less than 10 000, or a ‘hamlet’ which are places with fewer than 100-200 inhabitants and with little infrastructure (<https://wiki.openstreetmap.org/wiki/Key:place>). For our study areas in Congo and Gabon there were only 30 and 14 villages located in OpenStreetMap, respectively. Thus, we used satellite images from Google Earth to locate villages by the naked eye. In the end, we had 2,602 points for Guinée forestière, 335 points for our area in Congo and 100 points for our area in Gabon.

**Table A**. Sources of geographical data collected.

| **Type of data** | **Institution** | **Website** | **Reference** |
| --- | --- | --- | --- |
| *Species distribution* | IUCN | http://[www.iucnredlist.org/technicaldocuments/spatial-data](http://www.iucnredlist.org/technicaldocuments/spatial-data) | Reference according to species |
| *Forest cover* | FAO | http://www.fao.org/geonetwork/srv/en/main.home?uuid=ba4526fd-cdbf-4028-a1bd-5a559c4bff38 | [1] |
| *Cropland* |  |  |  |
| *Loss of forest cover* | University of Maryland | http://earthenginepartners.appspot.com/science-2013-global-forest/download_v1.6.ht | [2] |
| *Rainfall* | CHIRPS, University of California Santa Barbara | ftp://ftp.chg.ucsb.edu/pub/org/chg/products/CHIRPS-2.0 | [3] |
| *NDVI** | NASA | http://[modis.gsfc.nasa.gov/data/dataprod/mod13.php](https://modis.gsfc.nasa.gov/data/dataprod/mod13.php) | [4] |
| *Land surface temperature* | NASA | https://modis.gsfc.nasa.gov/data/dataprod/mod11.php | [5] |
| *Countries administrative boundaries* | OCHA (ONU) | [https://data.humdata.org/dataset/](https://data.humdata.org/dataset/guinea-geodatabase) | [6] |
| *Population density* | Afripop | [https://www.worldpop.org](https://www.worldpop.org/) | [7] |
| *Rivers* | NA | <https://www.diva-gis.org/gdata> | [8] |
| *Roads* | OpenStreetMap | <https://data.humdata.org/dataset/guinea-road-network>  <https://data.humdata.org/dataset/congo-roads> | [9] |
| *Villages* | OpenStreetMap; Google Earth | https://openstreetmap.org | [9,10] |

*Normalized Difference Vegetation Index

**Table B.** Identified risk factors and their associated variables. The spatial and temporal resolutions refer to the resolution of the collected spatial layers before manipulation.

| ***Risk factor*** | ***Associated variable*** | ***Spatial resolution*** | ***Temporal resolution*** |
| --- | --- | --- | --- |
| *Species distribution** | Binomial for presence/absence of each species | NA | Last estimation between 2008 et 2016, depending on species |
| *Forest cover* | % forest cover | 1 km | Estimate of % of land cover from several databases dating from 1998 to 2012 |
| *Cropland* | % cropland |  |  |
| *Cropland to forest ration* | % cropland / (% cropland + % forest cover) |  |  |
| *Loss of forest cover* | Binomial for loss of forest cover | 30 m | Loss of forest cover between 2001 and 2012 |
| *Landscape productivity* | NDVI | 1 km | Monthly |
| *Distance to rivers** | Euclidean distance | NA | NA |
| *Distance to roads** | Euclidean distance | NA | NA |
| *Human population density* | Number of persons per pixel | 100 m | Yearly |
| *Annual temperature range* | Maximal (Tmax) and minimal (Tmin) temperatures | 1 km | Tmax et Tmin from 8-day land surface temperature |
| *Mean annual temperature* | Mean annual temperature | 1 km | 8-day land surface temperature |
| *Mean monthly rainfall* | Mean monthly rainfall | 5 km | Monthly |
| *Bushmeat hunting areas** | Classed forests (Guinea). Areas under hunting pressure (Congo and Gabon) | NA | NA |
| *Bushmeat trade** | Euclidean distance to villages | NA | NA |
| *Presence of domestic animals** | Euclidean distance to villages | NA | NA |

* Shapefile transformed to raster

**Table C**. Geographical data manipulation to obtain risk factors data layers. (For original resolution of downloaded data see Table B).

| **Downloaded data** | **GIS Manipulation** | **Risk factor raster layer** |
| --- | --- | --- |
| Distribution for each species (polygon) | Reclassification- 0 : absence, 1 : presence | Species presence/absence |
| % of forest cover | NA | Forest cover |
| % of cropland | NA | Cropland |
|  | $Ratio=\% cropland / (\% cropland + \% forest cover)$ | Cropland to forest cover ratio |
| Loss or not (0, 1) of forest cover between 2001-2012 | Merging of tiles covering the study area; aggregation (mean) to a 1 km resolution* | Proportion of forest cover loss between 2001-2012 |
| NDVI^+^ (HDF file) | Conversion of HDF to Geotiff ; merging of tiles covering the study area; multiplication by 0.0001 [4] | NDVI |
| Rivers (polylines) | Estimation of Euclidean distance (in km) to rivers* | Distance to rivers |
| Roads (polylines) | Estimation of Euclidean distance (in km) to roads* | Distance to roads |
| Number of persons per pixel | Raster aggregation (mean) to a 1km resolution | Human density population |
| 8-day land surface temperature (HDF file, Kelvin degrees) | Conversion of HDF to Geotiff; merging of tiles covering the study area; multiplication by 0.02 [5]; conversion to Celsius = T - 273.15. Extraction of max and min temperatures. Estimation of *Annual temperature range* = Tmax-Tmin | Annual temperature range |
| 8-day land surface temperature (HDF file, Kelvin degrees) | Conversion of HDF to Geotiff ; merging of tiles covering the study area; multiplication by 0.02 [5]; conversion to Celsius = T - 273.15; mean calculation | Annual mean temperature |
| Mean monthly rainfall | Disaggregation (bilinear) to a 1 km resolution* | Mean monthly rainfall |

**Table C** *Continued.*

| Areas under hunting pressure | For Guinée forestière, georeferencing of polygons from maps; reclassification (1: forest, 0: everything else [11].  For Congo and Gabon areas, georeferencing of polygons from maps of hunting pressure in Central Africa according to [12]. The hunting areas have been reclassified from 0 to 4, areas under 1: low hunting pressure, 2: moderate pressure, 3: strong pressure, 4: strong pressure, probably overexploited, 0: everything else [12]. | Hunting areas |
| --- | --- | --- |
| Localization of villages (points) | Reclassification (1: village, 0: everything else); Euclidean distance calculation (km);  calculation of the inverse of the distance = [1 - [(village distance - MIN) / (MAX- MIN)]] * (MAX - MIN) + MIN | Inverse of the distance to villages |

^+^Normalized Difference Vegetation Index

* These raster layers had a difference of < 0.01 compared to the rest of the raster layers. Thus they were resampled (bilinear method) so that they would have the same pixel size as the rest.

**Table D** and **Fig A.** Fuzzy membership functions applied to the EBOV spillover risk factors.


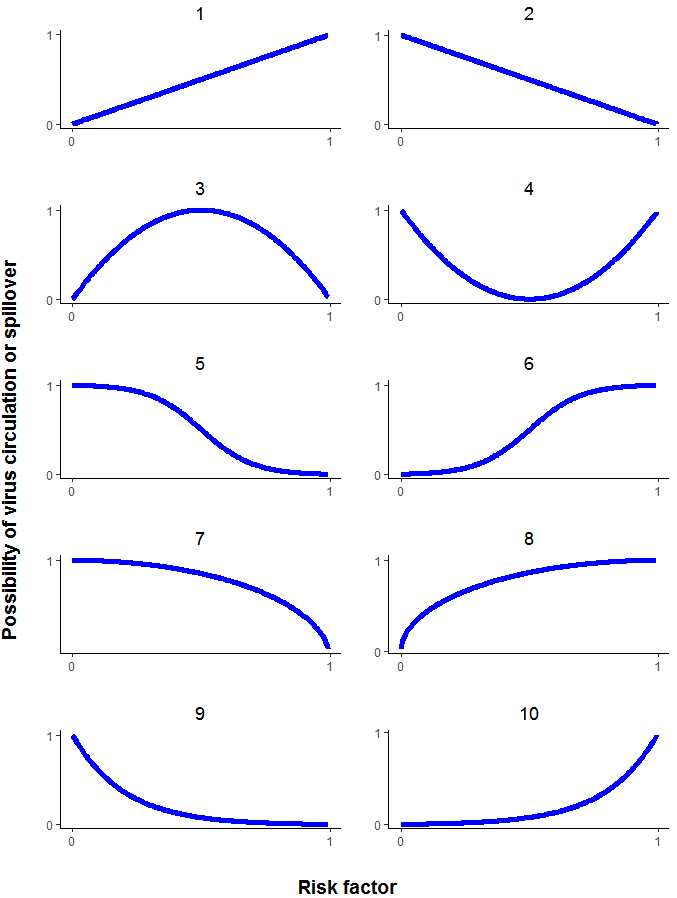


| **Risk factor** | **Function** |
| --- | --- |
| Forest cover | 3 |
| Cropland | 3 |
| Cropland to forest cover ratio | 3 |
| Loss of forest cover | 3 |
| Landscape productivity | 3 |
| Distance to rivers | 7 |
| Distance to roads | 2 |
| Human population density associated to the contact of potential reservoir or hosts of EBOV | 3 |
| Annual temperature range | 5 |
| Annual mean temperature | 1 |
| Mean monthly rainfall | 6 |
| Bushmeat hunting areas | 1 |
| Bushmeat trade | 1 |
| Presence of domestic animals | 8 |
| Human density population associated to bushmeat trade and hunting | 1 |

Choice of fuzzy functions:

- **Forest cover**: higher favorability values for *Ebolavirus* were associated with forested areas [13] and a greater forest cover was found in the surroundings of centers of first infection [14], but increasing maximum greening vegetation factor (MGVF) corresponded to decreasing risk [15]. We assumed that EBOV spillover is more likely at intermediate values of forest cover, where the probability of contact between wild animals and humans is more likely.
- **Cropland:** we considered that EBOV spillover is more likely at intermediate values of cropland because some crops can provide food for wildlife, particularly species of fruit bats (e.g. mango trees), making contact with humans and other species more likely. However, a landscape in which crops dominate, the presence of wild mammals decreases.
- **Cropland to forest cover ratio**: areas of higher favorability for Ebola virus were associated to mosaics of vegetation and crops [13]. Areas where a combination of forest and crops exists, as in agroforests, provide more opportunity for inter-species contact, including humans, than areas that are dominated by one of the land uses [16].
- **Loss of forest cover**: a higher proportion of dense forest loss favored EBOV outbreaks [17], and centers of first infection were located in areas with a higher degree of forest fragmentation [14]. Dense forests provide a protective effect against *Ebolavirus* spillover [15], whereas extreme deforestation leads to wildlife depletion.
- **Landscape productivity**: EBOV favorability was positively associated with enhanced vegetation index [18], and areas of marked NDVI anomalies preceded *Ebolavirus* outbreaks [19,20]. Intermediate levels of landscape productivity might promote a relative competition for food among species, leading to increased contact across species. In contrast, such contact might be diminished in less productive conditions or in scenarios of food abundance.
- **Distance to rivers**: proximity to water is an important factor for the presence of many wild mammals, and particularly of bats [21]; however, because of animal mobility, it is not a limiting factor but in large distances. Therefore, we considered that the probability of EBOV spillover would decrease asymptotically.
- **Distance to roads**: there was a negative correlation between presence of *Ebolavirus* in humans and distance to roads [13].
- **Human population density associated to the contact of potential reservoir or hosts of EBOV**: increasing population density has been associated with an increase in the risk of *Ebolavirus* spillover [18], as well as with a decrease in risk [15]. Contact with wildlife, and thus the probability of spillover would be higher at intermediate population densities.
- **Annual temperature range**: constant temperature favored *Ebolavirus* presence [13]. We considered that spillover would be more likely when temperature shows little annual variation, and that this probability rapidly decreases as variation in temperatures become more important.
- **Annual mean temperature**: We considered that higher mean annual temperatures are more favorable for mammal hosts, parasites and their interactions in tropical forests.
- **Mean monthly rainfall**: risk of *Ebolavirus* spillover has been associated with the transition from the dry to the wet season [18-20], which is reflected by a sigmoid function.
- **Bushmeat hunting areas and bushmeat trade**: EBOV spillover events were positively associated with bushmeat contact [22].
- **Presence of domestic animals**: serology tests have shown that antibodies against Ebola virus are present in domestic animals [23] and they could potentially be intermediate hosts for the virus. Thus, we assumed that the risk of EBOV spillover would increase asymptotically with the presence of domestic animals.
- **Human density population associated to bushmeat trade and hunting**: the activities associated to hunting, commercializing and consuming bushmeat are in general considered as risk factors for EBOV spillover. Therefore, we assumed that increasing population participating in these activities would be more at risk of being infected by the virus.

**Fig B**. Risk factors for EBOV spillover in Guinée forestière. A) Environmental, B) Climatic, C) Associated with bushmeat consumption, D) Potential reservoir / host species (dataset: doi:10.18167/DVN1/FZANMS).
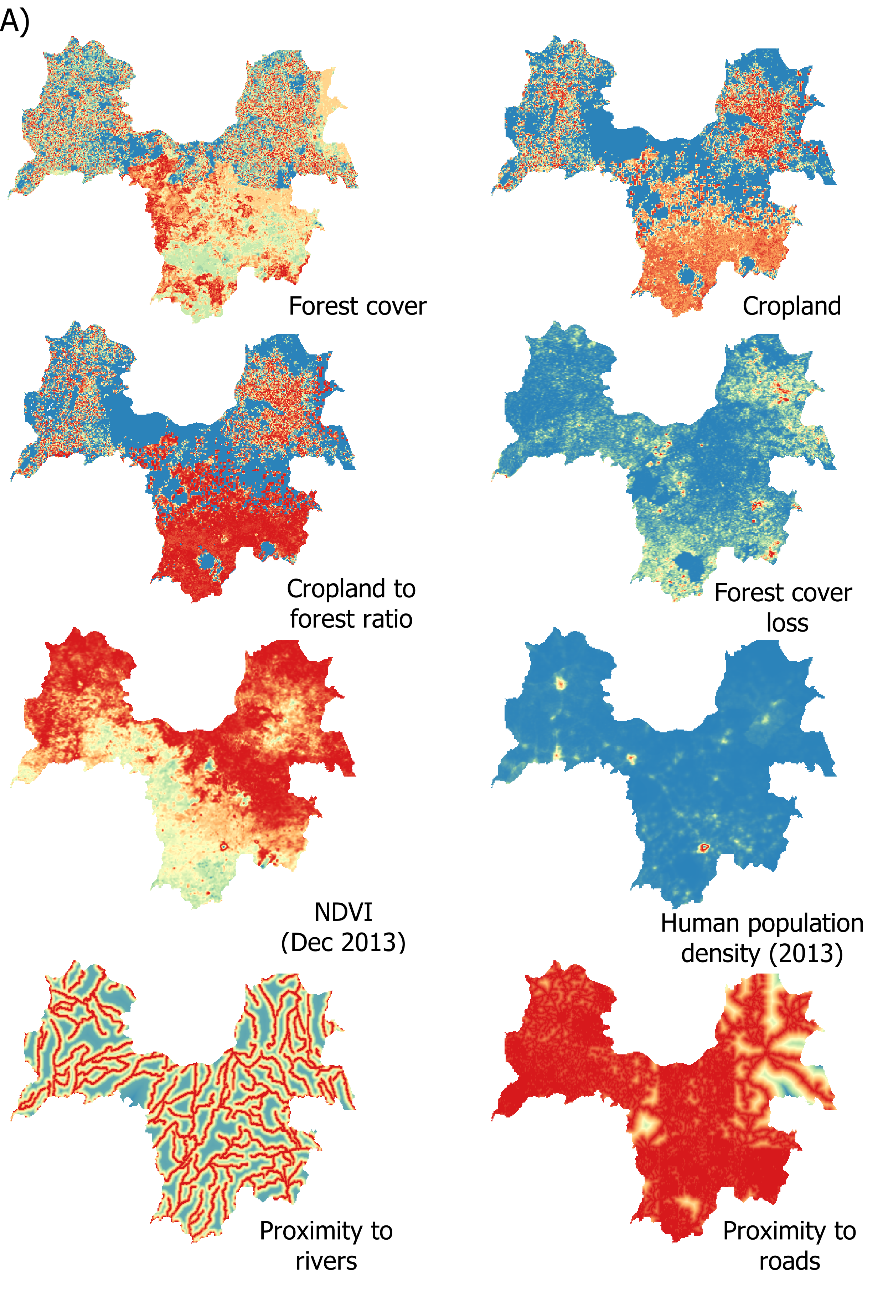


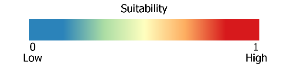

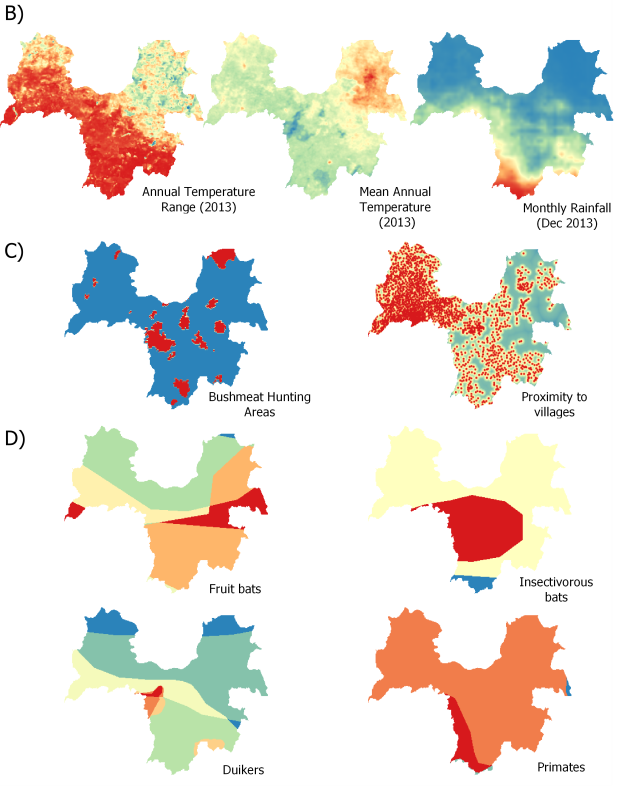


**Fig C.** Risk factors for EBOV spillover in the study area in Congo. A) Environmental, B) Climatic, C) Associated with bushmeat consumption, D) Potential reservoir / host species (dataset: doi:10.18167/DVN1/FZANMS).


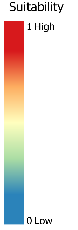

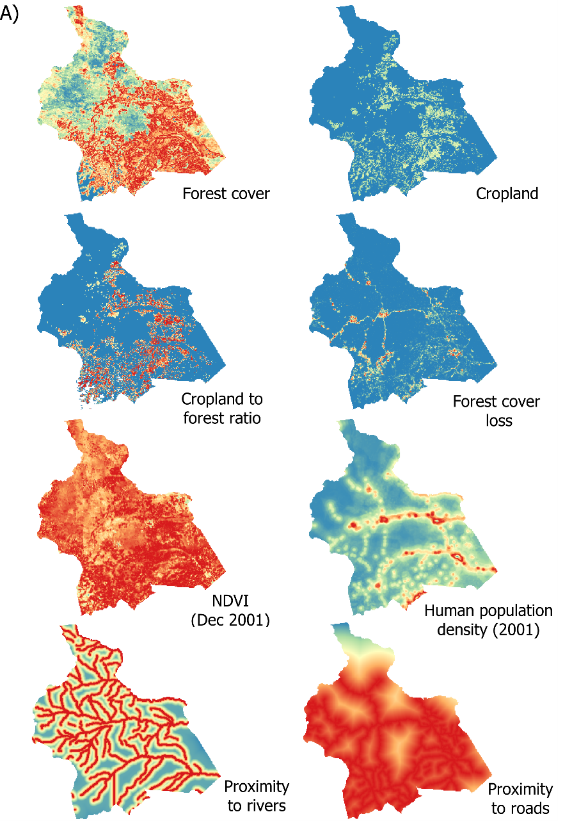


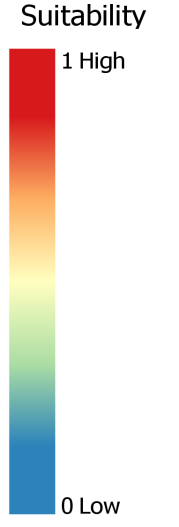

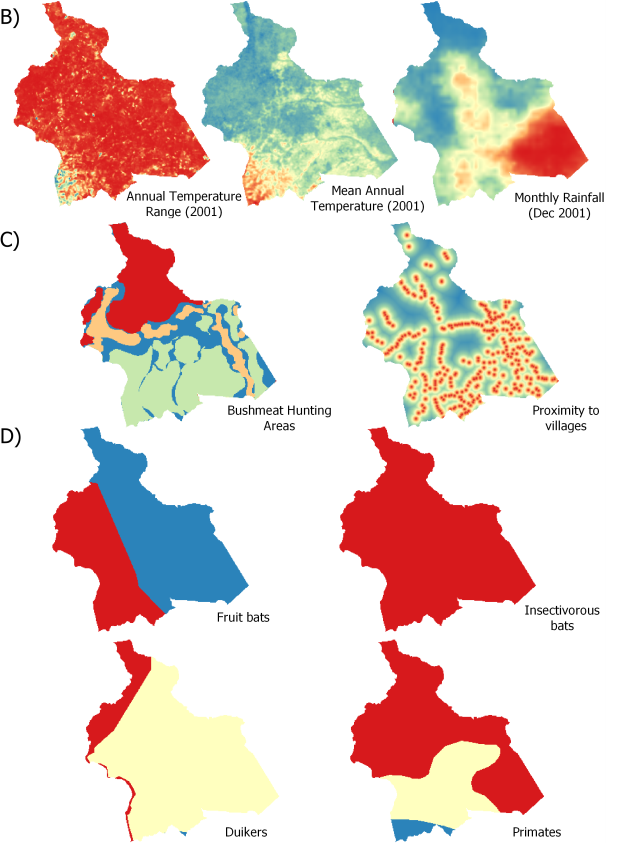


**Fig D.** Risk factors for EBOV spillover in the study area in Gabon. A) Environmental, B) Climatic, C) Associated with bushmeat consumption, D) Potential reservoir / host species (dataset: doi:10.18167/DVN1/FZANMS).
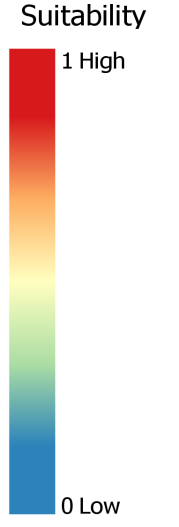

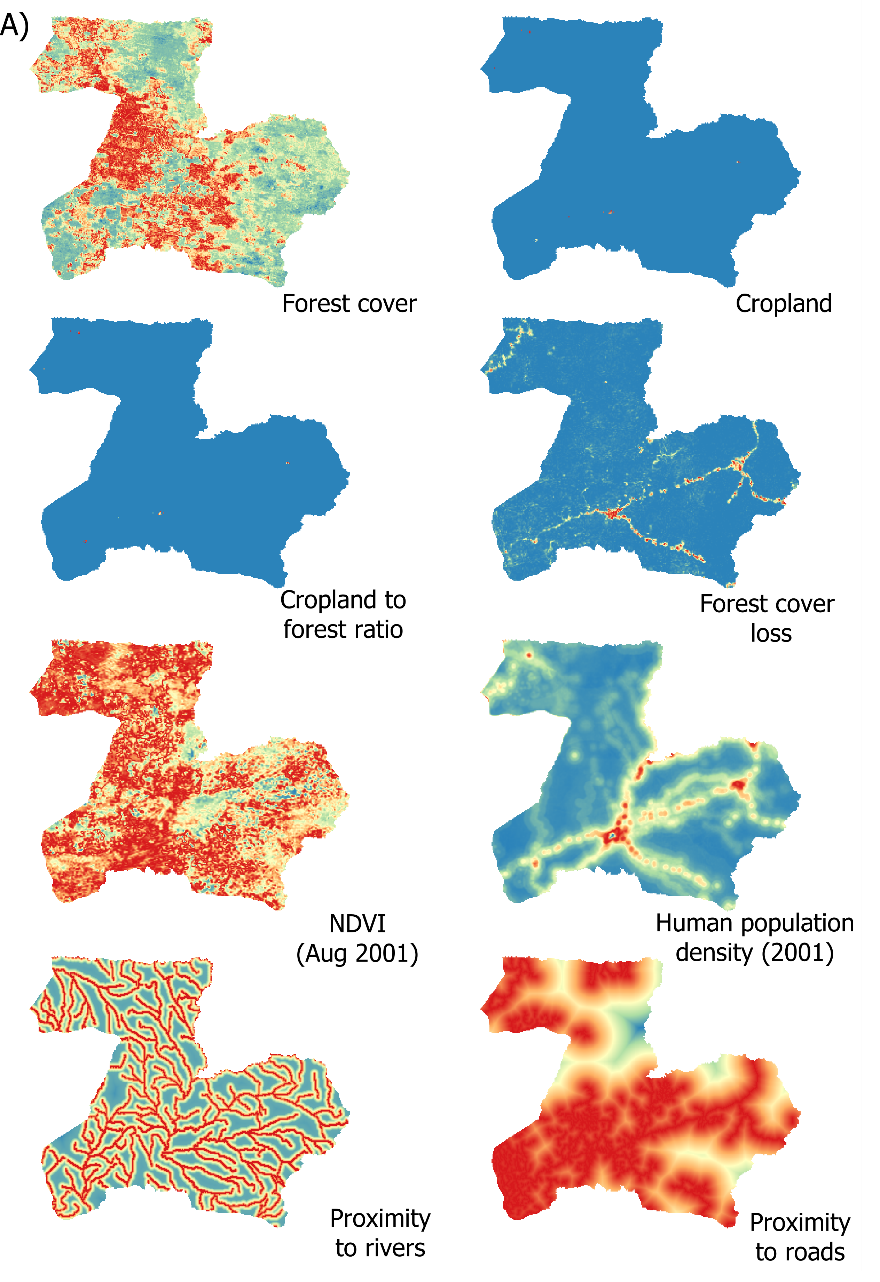


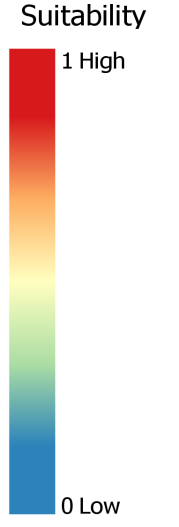

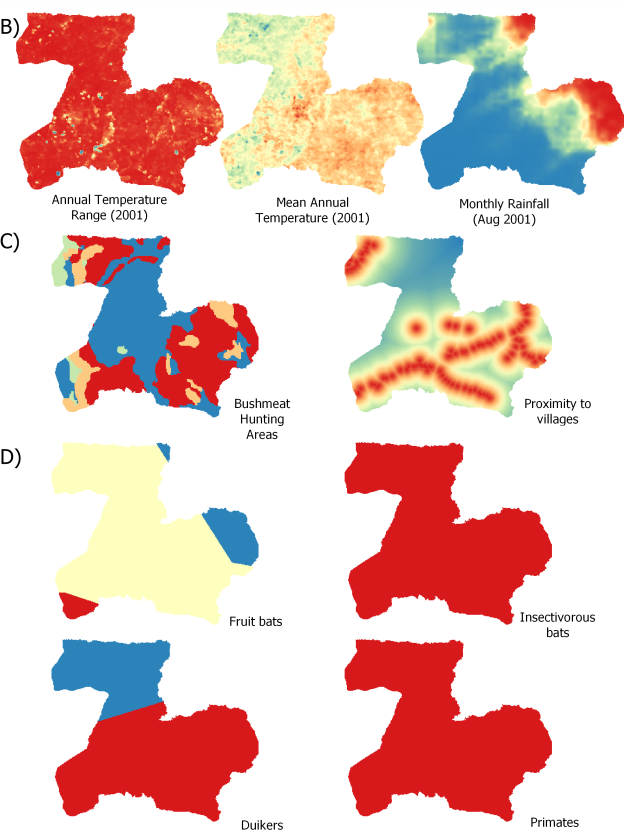


**References:**

1. Latham J, Cumani R, Rosati I, Bloise M. FAO Global Land Cover (GLC-SHARE) Database Beta-Release 1.0, Land and Water Division. 2014. Available from: http://www.fao.org/uploads/media/glc-share-doc.pdf
2. Hansen MC, Potapov PV, Moore R, Hancher M, Turubanova SA, Tyukavina A, et al. High-Resolution Global Maps of 21st-Century Forest Cover Change. Science. 2013; 342: 850–853. doi: 10.1126/science.1244693
3. Funk CC, Peterson PJ, Landsfeld MF, Pedreros DH, Verdin JP, Rowland JD, et al*.* A quasi-global precipitation time series for drought monitoring. *USGS*. 2014. Available from: https://pubs.usgs.gov/ds/832/pdf/ds832.pdf
4. Didan K, Barreto Munoz A, Solano R, Huete, A. MODIS Vegetation Index User’s Guide (MOD13 Series). Version 3.00. Vegetation Index and Phenology Lab, University of Arizona. 2015. Available from: https://lpdaac.usgs.gov/documents/103/MOD13_User_Guide_V6.pdf
5. Wan ZS, Hook G, Hulley G. MOD11A2 MODIS/Terra Land Surface Temperature/Emissivity 8-Day L3 Global 1km SIN Grid V006. Distributed by NASA EOSDIS Land Processes DAAC. 2015. doi: 10.5067/MODIS/MOD11A1.006
6. OCHA 2019. Humanitarian Data Exchange v1.33.1. Available from: https://data.humdata.org/.
7. Linard C, Gilbert M, Snow RW, Noor AM, Tatem AJ. Population Distribution, Settlement Patterns and Accessibility across Africa in 2010. *PLoS ONE.* 2012; 7: e31743. doi:10.1371/journal.pone.0031743
8. Hijmans R, Guarino L, Mathur P. DIVA-GIS. 2012. Available from: https://diva-gis.org/docs/DIVA-GIS_manual_7.pdf
9. OpenStreet Map. 2019. Map data copyrighted OpenStreetMap contributors and available from: https://www.openstreetmap.org
10. Google Earth. Image Landsat / Copernicus. Data SIO, NOAA, US Navy, NGA, GEBCO.
11. Direction Nationale des Eaux et Forêts & US Fish and Wildlife Service. Stratégie Nationale de gestion des éléphants en République de Guinée. 44 (2008). Available from: https://docplayer.fr/25958979-Direction-nationale-des-eaux-et-forets-us-fish-and-wildlife-service-ministere-de-l-agriculture-de-l-elevage-de-l-environnement-des-eaux-et-forets.html
12. Ziegler S, Fa JE, Wohlfart C, Streit B, Jacob S, Wegmann M. Mapping Bushmeat Hunting Pressure in Central Africa. Biotropica. 2016; 48: 405–412.
13. Olivero J, Fa JE, Real R, Farfán MÁ, Márquez AL, Vargas JM, et al. Mammalian biogeography and the Ebola virus in Africa. Mammal Rev. 2017; 47: 24–37. doi:10.1111/mam.12074.
14. Rulli MC, Santini M, Hayman DTS, D’Odorico P. The nexus between forest fragmentation in Africa and Ebola virus disease outbreaks | Sci Rep. 2017; 7: 41613. doi: 10.1038/srep41613.
15. Walsh MG, Haseeb MA. The landscape configuration of zoonotic transmission of Ebola virus disease in West and Central Africa: interaction between population density and vegetation cover. PeerJ. 2015; 3: e735. doi:10.7717/peerj.735.
16. Alexander KA, Sanderson CE, Marathe M, Lewis BL, Rivers CM, Shaman J, et al. What Factors Might Have Led to the Emergence of Ebola in West Africa? PLoS Neglect Trop D. 2015; 9: e0003652. doi:10.1371/journal.pntd.0003652.
17. Olivero J, Fa JE, Real R, Márquez AL, Farfán MA, Vargas JM, et al. Recent loss of closed forests is associated with Ebola virus disease outbreaks. Sci Rep.2017; 7: 14291. doi:10.1038/s41598-017-14727-9.
18. Schmidt JP, Park AW, Kramer AM, Han BA, Alexander LW, Drake JM. Spatiotemporal Fluctuations and Triggers of Ebola Virus Spillover. Emerg Infect Dis. 2017; 23: 415–422. doi:10.3201/eid2303.160101.
19. Pinzon JE, Wilson JM, Tucker CJ, Arthur R, Jahrling PB, Formenty P. Trigger events: enviroclimatic coupling of Ebola hemorrhagic fever outbreaks. Am J Trop Med Hyg. 2004; 71: 664–674. doi:10.4269/ajtmh.2004.71.664.
20. Tucker CJ, Wilson JM, Mahoney R, Anyamba A, Linthicum K, Myers MF. Climatic and ecological context of the 1994-1996 Ebola outbreaks. Photogramm Eng Remote Sensing. 2002; 68: 147–152.
21. Herkt KMB, Barnikel G, Skidmore AK, Fahr J. A high-resolution model of bat diversity and endemism for continental Africa. Ecol Model. 2016; 320: 9–28. doi:10.1016/j.ecolmodel.2015.09.009.
22. Judson SD, Fischer R, Judson A, Munster VJ. Ecological Contexts of Index Cases and Spillover Events of Different Ebolaviruses. PLOS Pathog. 2016; 12: e1005780. doi:10.1371/journal.ppat.1005780.
23. Fischer K, Camara A, Troupin C, Fehling SK, Strecker T, Groschup MH et al. Serological evidence of exposure to Ebolaviruses in domestic pigs from Guinea. Transbound Emerging Dis. 2020; 67: 724-32. doi:10.1111/tbed.13391.
