# Supplementary material for: Mapping of Ebola virus spillover: Suitability and seasonal variability at the landscape scale
Source: PLoS Negl Trop Dis. 2021 Aug 23;15(8):e0009683. doi: 10.1371/journal.pntd.0009683 (PMC8425568; doi:10.1371/journal.pntd.0009683)
Supplement: S2 Text — (DOCX) [file pntd.0009683.s003.docx]

**S2 Text. Assessment of potential reservoir and intermediate host species**

We focused on fruit bats, insectivorous bats, primates and duikers. Bats have been signaled previously as potential reservoirs for *Ebolavirus* [1-3], whereas primates and duikers have been implicated in previous *Ebolavirus* spillover events [4]. We considered fruit bats and insectivorous bats separately because their interaction with humans for a spillover of the virus to occur probably differs. To assess the relative importance of species we assigned a value from 1 to 5 according to the likelihood of the species of being a potential reservoir or intermediate host of the virus.

For bat species, we gave more weight to species where RNA sequences of *Ebolavirus* have been found, followed by species that have tested positive to virus antibodies [2,5-8].

Spillover of *Ebolavirus* from primates and duikers to humans is likely related to species that are susceptible to infection and that can be hunted or found in the forest and consumed by humans. Among duiker species, we considered the bay duiker (*Cephalophus dorsalis*) as more important than other species because a carcass positive for the virus has been previously reported only for this species [9].

Among primates, we considered gorillas, chimpanzees and greater spot-nosed monkeys (*Cercopithecus nictitans*), as all these species have been associated to *Ebolavirus* infection [4,10]. Gorillas and chimpanzees were considered much more important than the spot-nosed monkey because they have tested positive for *Ebolavirus* [9], they have been reported as potential sources of spillover [4] and high mortality rates on these species have been associated with Ebola infection [11-13].

Species considered in this study and their relative importance for *Ebolavirus* spillover are shown in Table A.

We considered that the four groups of species differ in their relevance for spillover, thus we carried pairwise comparisons between them using a pairwise matrix to generate the weight for each group. We considered both groups of bats of equal importance and more important in general than primates and duikers for the spillover of the virus to human. Primates and duikers were considered equally important.

To produce the suitability map of species, we first created a map for each group of species that took into account the relative importance given to each species (Table A). Each of these four maps was then multiplied by its corresponding weight before being combined to produce the final species map.

**Table A.** Species considered as potential reservoir or intermediate host species of *Ebolavirus* and the relative importance given to each species.

| **Species** | **Relative importance** | **Species** | **Relative importance** |
| --- | --- | --- | --- |
| ***Fruit bats*** |  | ***Duikers*** |  |
| *Eidolon helvum* | 3 | *Cephalophus dorsalis* | 5 |
| *Epomophorus gambianus* | 3 | *Cephalophus jentinki* | 3 |
| *Epomophorus labiatus* | 3 | *Cephalophus niger* | 3 |
| *Epomophorus wahlbergi* | 3 | *Cephalophus ogilbyi* | 3 |
| *Epomops franqueti* | 5 | *Cephalophus rufilatus* | 3 |
| *Hypsignathus monstrosus* | 5 | *Cephalophus silvicultor* | 3 |
| *Lissonycteris angolensis* | 3 | *Cephalophus zebra* | 3 |
| *Micropteropus pusillus* | 3 | *Philantomba maxwellii* | 3 |
| *Myonycteris torquata* | 5 | *Cephalophus callipygus* | 3 |
| *Rousettus aegyptiacus* | 3 | *Cephalophus leucogaster* | 3 |
|  |  | *Cephalophus nigrifrons* | 3 |
| ***Insectivorous bats*** |  | *Philantomba monticola* | 3 |
| *Chaerephon pumilus* | 3 |  |  |
| *Miniopterus inflatus* | 3 | ***Primates*** |  |
| *Mops condylurus* | 5 | *Cercopithecus nictitans* | 1 |
| *Otomops martiensseni* | 3 | *Pan troglodytes* | 5 |
|  |  | *Gorilla gorilla* | 5 |

References

1. Goldstein T, Anthony SJ, Gbakima A, Bird BH, Bangura J, Tremeau-Bravard A, et al. The Discovery of Bombali Virus Adds Further Support for Bats as Hosts of Ebolaviruses. Nat Microbiol. 2018; 3: 1084–89. doi:10.1038/s41564-018-0227-2.
2. Leroy EM., Kumulungui B, Pourrut X, Rouquet P, Hassanin A, Yaba P, et al. Fruit Bats as Reservoirs of Ebola Virus. Nature. 2005; 438: 575. doi: 10.1038/438575a.
3. Leroy EM, Epelboin A, Mondonge V, Pourrut X, Gonzalez JP, Muyembe-Tamfum JJ, et al. Human Ebola Outbreak Resulting from Direct Exposure to Fruit Bats in Luebo, Democratic Republic of Congo, 2007. Vector-Borne Zoonot. 2009; 9: 723–728. doi:10.1089/vbz.2008.0167.
4. Judson SD, Fischer R, Judson A, Munster VJ. Ecological Contexts of Index Cases and Spillover Events of Different Ebolaviruses. PLOS Pathog. 2016; 12: e1005780. doi:10.1371/journal.ppat.1005780.
5. Olival KJ, Hayman DTS. Filoviruses in Bats: Current Knowledge and Future Directions. Viruses. 2014; 6:1759–1788.
6. De Nys HM, Kingebeni PM, Keita AK, Butel C, Thaurignac C, Villabona-Arenas CJ, et al. Survey of Ebola Viruses in Frugivorous and Insectivorous Bats in Guinea, Cameroon, and the Democratic Republic of the Congo, 2015–2017.  Emerg Infect Dis. 2018; 24: 2228–2240. doi:10.3201/eid2412.180740.
7. Swanepoel R, Leman PA, Burt FJ, Zachariades NA, Braack LE, Ksiazek TG, et al. Experimental inoculation of plants and animals with Ebola virus. Emerg Infect Dis. 1996; 2: 321-25.
8. Kupferschmidt K. This bat species may be the source of the Ebola epidemic that killed more than 11,000 people in West Africa. Science. 2019. Available from: https://www.sciencemag.org/news/2019/01/bat-species-may-be-source-ebola-epidemic-killed-more-11000-people-west-africa. (Accessed: 16th May 2019).
9. Rouquet P, Froment JM, Bermejo M, Kilbourn A, Karesh W, Reed P, et al. Wild Animal Mortality Monitoring and Human Ebola Outbreaks, Gabon and Republic of Congo, 2001–2003. Emerg Infect Dis. 2005; 11:283‑90. doi: 10.3201/eid1102.040533.
10. Boumandouki P, Formenty P, Epelboin A, Campbell P, Atsangandoko C, Allarangar Y, et al. Clinical management of patients and deceased during the Ebola outbreak from October to December 2003 in Republic of Congo. Bulletin de la Societe de pathologie exotique. 2005; 98: 218–23.
11. Huijbregts B, Wachter PD, Obiang LSN, Akou ME. Ebola and the Decline of Gorilla Gorilla Gorilla and Chimpanzee Pan Troglodytes Populations in Minkebe Forest, North-Eastern Gabon. Oryx. 2003; 37: 437–43. doi:10.1017/S0030605303000802.
12. Leroy EM, Rouquet P, Formenty P, Souquière S, Kilbourne A, Froment JM, et al. Multiple Ebola Virus Transmission Events and Rapid Decline of Central African Wildlife. Science. 2004; 303: 387–90.doi: 10.1126/science.1092528.
13. Lahm SA, Kombila, M., Swanepoel R, Barnes RFW. Morbidity and mortality of wild animals in relation to outbreaks of Ebola haemorrhagic fever in Gabon, 1994–2003. Transactions of The Royal Society of Tropical Medicine and Hygiene. 2007; 101, 64–78.
