# Supplementary material for: Mapping of Ebola virus spillover: Suitability and seasonal variability at the landscape scale
Source: PLoS Negl Trop Dis. 2021 Aug 23;15(8):e0009683. doi: 10.1371/journal.pntd.0009683 (PMC8425568; doi:10.1371/journal.pntd.0009683)
Supplement: S3 Text — (DOCX) [file pntd.0009683.s004.docx]

**S3 Text. GIS-MCE applied to produce suitability maps for *Ebolavirus* maintenance in fruit bats and insectivorous bats**

GIS-MCE was applied to map suitable areas for *Ebolavirus* maintenance in bats in Guinée forestière.

It is still unclear what factors and how such factors interact to influence the maintenance of *Ebolavirus* in bats. We therefore decided to use the same environmental and climatic risk factors that were used for suitability maps for EBOV spillover, with the exception of human density population, which was removed from the environmental category. We also removed all the risk factors related to bushmeat trade and consumption. Table A shows the risk factors considered.

Weights were estimated for each risk factor within their corresponding category (i.e. environmental, climatic, bat species) and pairwise comparisons between factors were done in a five-point scale (i. e. from strongly less important to strongly more important). It is likely that many of the climatic and environmental factors that influence *Ebolavirus* maintenance in bats will affect different bat species in different ways. However there are still important gaps in our knowledge not only on which bat species are part of the reservoir for *Ebolavirus*, but also on the role that climatic and environmental variables play on the physiology and therefore on the maintenance of the virus in bat populations. For this reason, we decided to use the same weights of risk factors that were used for EBOV spillover (see main text). Tables B and C show the pairwise comparisons for environmental and climatic factors, respectively.

We focused on fruit bats and insectivorous bats. As for the EBOV spillover suitability maps, we gave more weight to species where RNA sequences of *Ebolavirus* have been found, followed by species that have tested positive to virus antibodies (See S1 Text). Table D shows the bat species that were taken into account.

To validate the suitability maps for *Ebolavirus* maintenance in bats, we used data of bats sampled in December 2016 and March 2017 in Guinée forestière to look for the presence of *Ebolavirus* antibodies (De Nys *et al.*  2018). Four antigens were used in this study to evaluate the presence of antibodies against the virus, and four statistical methods to determine the cut-off value for each antigen. We used the least strict statistical method to asses if bats were positive or negative to have a larger dataset. To validate our maps, we compared the location of sampling sites where bats tested positive for at least one *Ebolavirus* (*i.e.* *Zaire*, *Sudan*, or *Bundibugyo* *Ebolavirus*) with the suitability maps produced. A sample was considered as positive if it came out positive for at least one of the three *Ebolavirus*. With this method, of 4,022 samples tested in the study, 734 came out positive to at least one *Ebolavirus* (De Nys *et al*. 2018). This translated to 34 positive bats (20 fruit bats and 14 insectivorous bats) from 129 (58 fruit bats and 71 insectivorous bats) that were sampled in our study area of Guinée forestière. We extracted the pixel value of sites where at least one bat tested positive to *Ebolavirus* antibodies to compare with the pixel value of sites where all sampled bats came out negative.

**Table A.** Factors associated with the risk of *Ebolavirus* maintenance in fruit bats and insectivorous bats.

|  | ***Risk factor*** |
| --- | --- |
| *Presence o potential maintenance bat species* | Species distribution |
| *Environmental factors* | Forest cover |
|  | Cropland |
|  | Cropland to forest ratio |
|  | Loss of forest cover* |
|  | Landscape productivity (i.e. NDVI) |
|  | Distance to rivers |
|  | Distance to roads |
| *Climatic factors* | Annual temperature range |
|  | Annual mean temperature |
|  | Mean monthly rainfall |

*Loss of forest cover was obtained from the Global Forest Change platform of the University of Maryland. In contrast with the loss of forest cover used for the suitability maps for EBOV spillover (see main text), here the downloaded data of loss of forest cover was encoded as 0, representing no forest loss, or a value between 1 and 17, representing the loss detected primarily in the year 2001 to 2018, respectively. All the non-zero values were reclassified to one, so that the final raster layer represented the proportion of forest cover loss between 2001 and 2018.

**Table B.** Pairwise comparisons of environmental factors associated with *Ebolavirus* maintenance in bats.

| Risk factor B  Risk factor A | *Forest cover* | *Cropland* | *Cropland to forest cover ratio* | *Loss of forest cover* | *Landscape productivity* | *Proximity to rivers* | *Proximity to roads* | **Weight** |
| --- | --- | --- | --- | --- | --- | --- | --- | --- |
| *Forest cover* | 1 | 5 | 3 | 1 | 3 | 5 | 5 | **0.292** |
| *Cropland* |  | 1 | 1/3 | 1/5 | 3 | 3 | 1 | **0.062** |
| *Cropland to forest cover ratio* |  |  | 1 | 1/3 | 1 | 3 | 3 | **0.126** |
| *Loss of forest cover* |  |  |  | 1 | 3 | 5 | 5 | **0.292** |
| *Landscape productivity* |  |  |  |  | 1 | 3 | 3 | **0.126** |
| *Proximity to rivers* |  |  |  |  |  | 1 | 1/3 | **0.039** |
| *Proximity to roads* |  |  |  |  |  |  | 1 | **0.062** |

**Table C.** Pairwise comparisons of climatic factors associated with *Ebolavirus* maintenance in bats.

| Risk factor B  Risk factor A | *Annual temperature range* | *Annual mean temperature* | *Mean monthly rainfall* | **Weight** |
| --- | --- | --- | --- | --- |
| *Annual temperature range* | 1 | 3 | 1/5 | **0.211** |
| *Annual mean temperature* |  | 1 | 1/5 | **0.102** |
| *Mean monthly rainfall* |  |  | 1 | **0.686** |

**Table D.** Species considered as potential reservoir species of *Ebolavirus* and the relative importance given to each species.

| **Species** | **Relative importance** | **Species** | **Relative importance** |
| --- | --- | --- | --- |
| ***Fruit bats*** |  | ***Insectivorous bats*** |  |
| *Eidolon helvum* | 3 | *Chaerephon pumilus* | 3 |
| *Epomophorus gambianus* | 3 | *Miniopterus inflatus* | 3 |
| *Epomophorus labiatus* | 3 | *Mops condylurus* | 5 |
| *Epomophorus wahlbergi* | 3 | *Otomops martiensseni* | 3 |
| *Epomops franqueti* | 5 |  |  |
| *Hypsignathus monstrosus* | 5 |  |  |
| *Lissonycteris angolensis* | 3 |  |  |
| *Micropteropus pusillus* | 3 |  |  |
| *Myonycteris torquata* | 5 |  |  |
| *Rousettus aegyptiacus* | 3 |  |  |
