# Supplementary material for: Mapping of Ebola virus spillover: Suitability and seasonal variability at the landscape scale
Source: PLoS Negl Trop Dis. 2021 Aug 23;15(8):e0009683. doi: 10.1371/journal.pntd.0009683 (PMC8425568; doi:10.1371/journal.pntd.0009683)
Supplement: S4 Text — (DOCX) [file pntd.0009683.s005.docx]

**S4 Text.**  **Pairwise comparison matrices of the analytical hierarchy process (AHP) for risk factors associated with EBOV spillover**

For all pairwise comparisons: 5- Risk factor A is strongly more important than risk factor B; 3- risk factor A is moderately more important than risk factor B; 1- risk factor A is equally important to risk factor B; 1/3- risk factor A is moderately less important than risk factor B; 1/5 risk factor A is strongly less important than risk factor B.

**Table A.** Pairwise comparisons of environmental factors associated with the risk of EBOV spillover.

| Risk factor B  Risk factor A | *Forest cover* | *Cropland* | *Cropland to forest cover ratio* | *Loss of forest cover* | *Landscape productivity* | *Proximity to rivers* | *Proximity to roads* | *Human population density* | **Weight** |
| --- | --- | --- | --- | --- | --- | --- | --- | --- | --- |
| *Forest cover* | 1 | 5 | 3 | 1 | 3 | 5 | 5 | 3 | **0.255** |
| *Cropland* |  | 1 | 1/3 | 1/5 | 3 | 3 | 1 | 1/5 | **0.048** |
| *Cropland to forest cover ratio* |  |  | 1 | 1/3 | 1 | 3 | 3 | 1/3 | **0.096** |
| *Loss of forest cover* |  |  |  | 1 | 3 | 5 | 5 | 3 | **0.255** |
| *Landscape productivity* |  |  |  |  | 1 | 3 | 3 | 1/3 | **0.096** |
| *Proximity to rivers* |  |  |  |  |  | 1 | 1/3 | 1/5 | **0.032** |
| *Proximity to roads* |  |  |  |  |  |  | 1 | 1/3 | **0.05** |
| *Human population density* |  |  |  |  |  |  |  | 1 | **0.167** |

**Table B.** Pairwise comparisons of climatic factors associated with EBOV spillover.

| Risk factor B  Risk factor A | *Annual temperature range* | *Annual mean temperature* | *Mean monthly rainfall* | **Weight** |
| --- | --- | --- | --- | --- |
| *Annual temperature range* | 1 | 3 | 1/5 | **0.211** |
| *Annual mean temperature* |  | 1 | 1/5 | **0.102** |
| *Mean monthly rainfall* |  |  | 1 | **0.686** |

**Table C.** Pairwise comparisons of factors associated with bushmeat trade and consumption with EBOV spillover.

| Risk factor B  Risk factor A | *Bushmeat hunting areas* | *Bushmeat trade* | *Human population density* | *Presence of domestic animals* | **Weight** |
| --- | --- | --- | --- | --- | --- |
| *Bushmeat hunting areas* | 1 | 1 | 3 | 5 | **0.380** |
| *Bushmeat trade* |  | 1 | 3 | 5 | **0.380** |
| *Human population density* |  |  | 1 | 5 | **0.179** |
| *Presence of domestic animals* |  |  |  | 1 | **0.062** |

**Table D.** Pairwise comparisons of the four groups of species considered as potential reservoirs or intermediate hosts for *Ebolavirus*.

| Risk factor B  Risk factor A | *Fruit bats* | *Insectivorous bats* | *Duikers* | *Primates* | **Weight** |
| --- | --- | --- | --- | --- | --- |
| *Fruit bats* | 1 | 1 | 3 | 3 | **0.375** |
| *Insectivorous bats* |  | 1 | 3 | 3 | **0.375** |
| *Duikers* |  |  | 1 | 1 | **0.125** |
| *Primates* |  |  |  | 1 | **0.125** |
