# Supplementary material for: Mapping of Ebola virus spillover: Suitability and seasonal variability at the landscape scale
Source: PLoS Negl Trop Dis. 2021 Aug 23;15(8):e0009683. doi: 10.1371/journal.pntd.0009683 (PMC8425568; doi:10.1371/journal.pntd.0009683)

**S5 Text.**  **Supplementary Results**

**Fig A.** Suitability maps of *Ebolavirus* maintenance in bats in December 2016 (A) and March 2017 (B) in Guinée forestière. Fruit bats and insectivorous bats were considered of equal importance (dataset: doi:10.18167/DVN1/FZANMS).


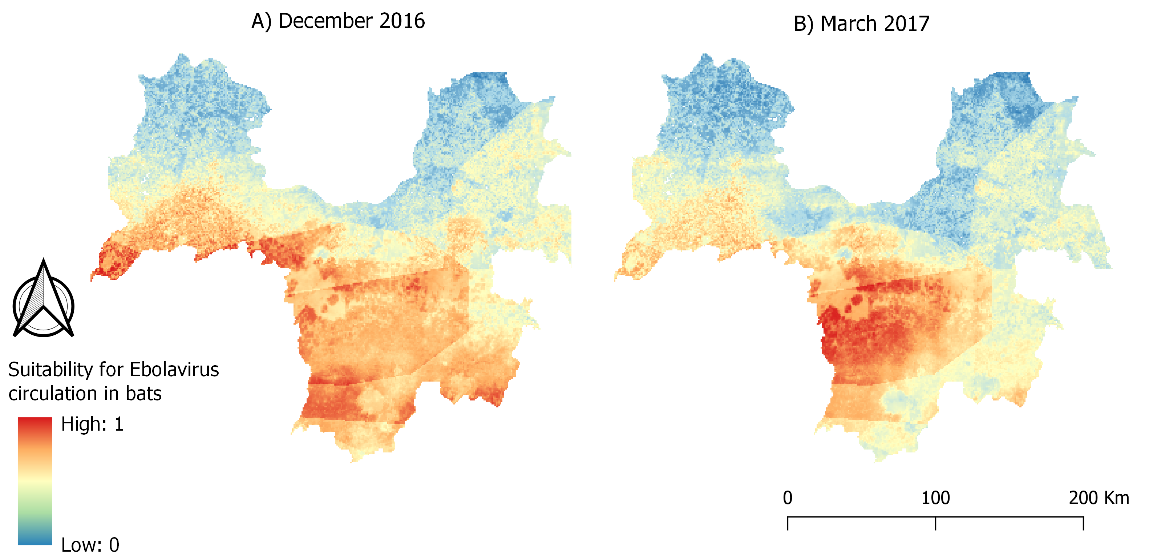


**Fig B.** Suitability maps of climatic factors and mean monthly rainfall for the study area in Congo (dataset: doi:10.18167/DVN1/FZANMS).


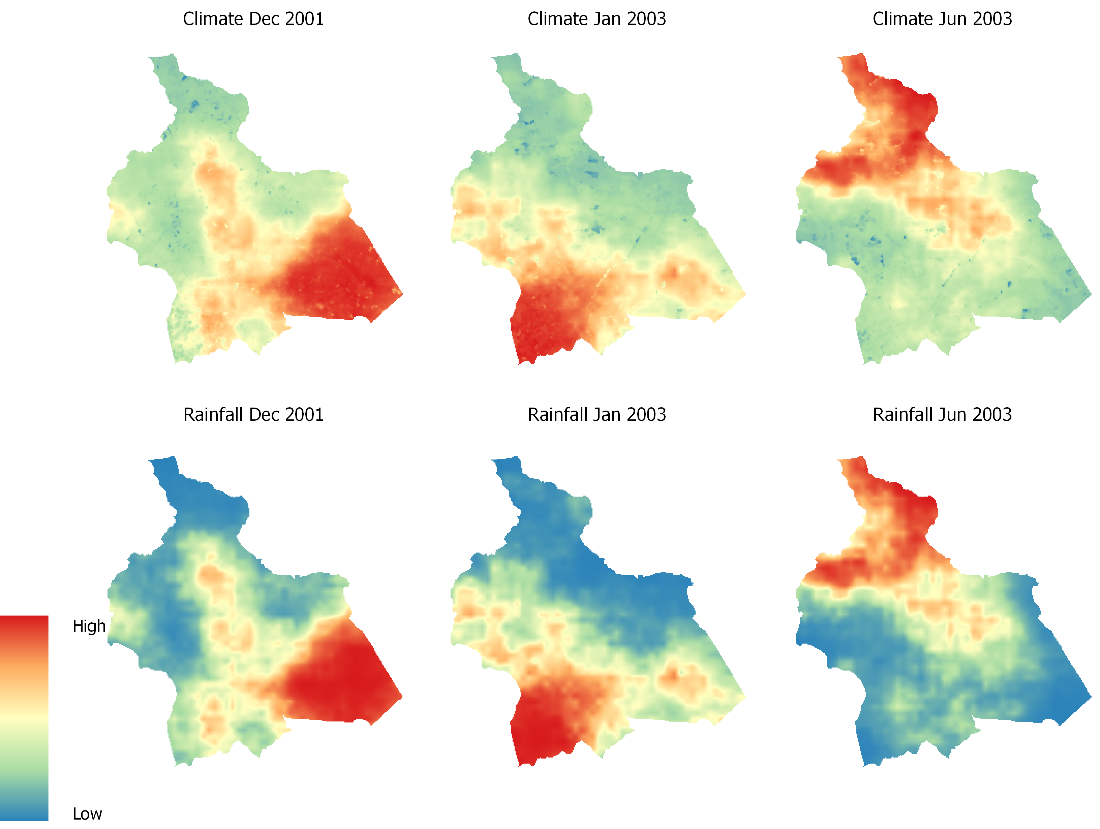


**Fig C.** Rainfall in Guinée forestière in 2013 (dataset: doi:10.18167/DVN1/FZANMS).


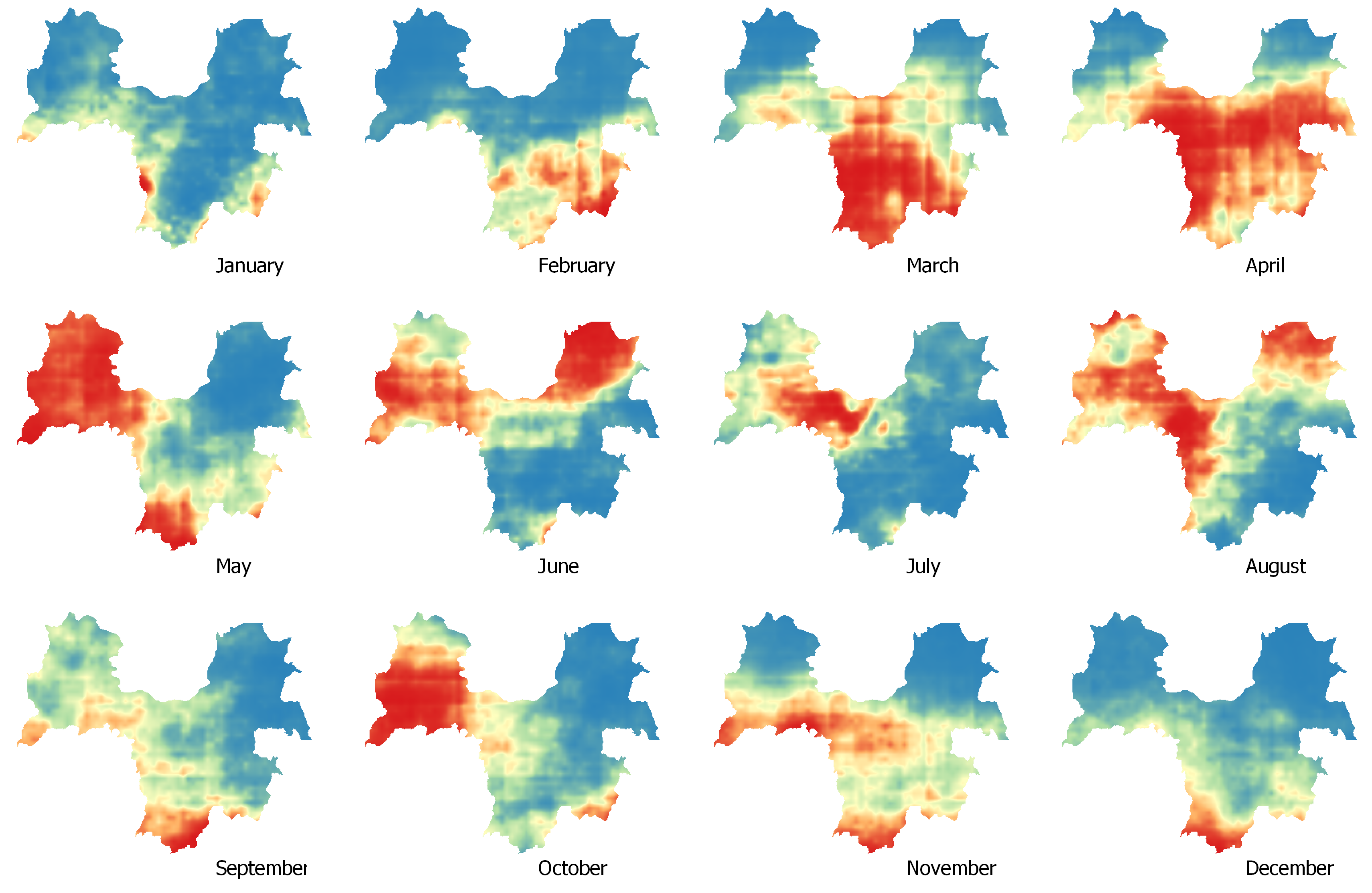

Supplement: S5 Text — (DOCX) [file pntd.0009683.s006.docx]
