# Supplementary material for: Mapping of Ebola virus spillover: Suitability and seasonal variability at the landscape scale
Source: PLoS Negl Trop Dis. 2021 Aug 23;15(8):e0009683. doi: 10.1371/journal.pntd.0009683 (PMC8425568; doi:10.1371/journal.pntd.0009683)
Supplement: S6 Text — (DOCX) [file pntd.0009683.s007.docx]

### AREA OF STUDY ####

## 1_AOI_script

## This script allows to create the spatial object from the shapefile for each area of interest (Guinea and Congo)

## Shapefiles were downloaded from

## https://data.humdata.org/dataset/guinea-geodatabase for Guinea

## https://data.humdata.org/dataset/congo-administrative-boundaries for Congo

### Create spatial object of the area of interest ####

## A shapefile of Forested Guinea was created from the shapefile of entire Guinea in ArcGis. This shapefile has 7 polygons, one for each prefecture in Forested Guinea.

## From the shapefile of Forested Guinea, create a Spatial object with the correct projection to be used in other functions

library(tidyverse)

library(sf)

library(sp)

library(ggplot2)

library(lwgeom)

library(raster)

wd="" #path to working directory MTD

setwd(wd)

FG<-read_sf(dsn="./Guinee/cartes_Guinee/Guinee_forestiere",layer="G_forestiere") #load shapefile of study area boundaries

ggplot(FG)+geom_sf()

#fusion all polygons into one

FG$area<-st_area(FG)

FG<-FG%>%

summarise(area=sum(area))

ggplot(FG)+geom_sf()

FG<-as_Spatial(FG,IDs="Forested_Guinea") #convert to Spatial object

proj_WGS84=CRS("+init=epsg:4326") #define projection

proj_Dab81=CRS("+init=epsg:3462") #projection for Guinea, Dabola 1981 / UTM zone 29N

FG_WGS84<-spTransform(FG,proj_WGS84) #Reprojection

FG_Dab81<-spTransform(FG,proj_Dab81) #Reprojection

Extent_WGS84<-extent(FG_WGS84) #create the extent area using the shape of the area of interest

rast_WGS84<-raster(ncol=369, nrows=301,ext=Extent_WGS84,crs=proj_WGS84) #create empty raster

Extent_Dab81<-extent(FG_Dab81) #create the extent area using the shape of the area of interest

rast_Dab81<-raster(ncol=369, nrows=301,ext=Extent_Dab81,crs=proj_Dab81) #create empty raster

## the resolution is aprox 1km (size of pixel).

save(Extent_Dab81, Extent_WGS84, FG, FG_Dab81,FG_WGS84,proj_Dab81,proj_WGS84,rast_Dab81,rast_WGS84, file="AOI.Rdata") #save to load at the beginning of other scripts, so that objects will be loaded to the working environment

###########################################################################################

#### FORMATING DATA FOR SPATIAL MULTICRITERIA EVALUATION ANALYSIS ####

library(raster)

library(rgdal)

library(sp)

library(rgeos)

library(gdalUtils)

## Data are various rasters downloaded from open access databases.

## Each raster corresponds to a variable associated with a risk factor of Ebola virus spillover

wd="/.../" #path to working directory where rasters are located, organized in different folders

setwd(wd)

load("AOI.Rdata") # has raster and extent of study area in Guinea

pathFD="/.../" #path to folder for saving formatted rasters

## Define projection and Extent####

## From Script 1.AOI the following objects are loaded

FG_WGS84 #shape of area of interest

Extent_WGS84 #extent of area of interest in WGS geog. coord. system

rast_WGS84 #raster of area of interest in WGS geog. coord. system

proj_Dab81=CRS("+init=epsg:3462") #projection for Guinea

Extent_Dab81#extent of study area in projection for Guinea

rast_Dab81 #raster of study area in projection for Guinea

### FOREST DATA ####

## Raster of percentage of forest cover; resolution 1km

GDALinfo(".\\Guinee\\Forest_Cover\\glc_shv10_04.tif") #to access metadata of raster

TFC<-raster(".\\Guinee\\Forest_Cover\\glc_shv10_04.tif") #load raster

TFC<-crop(TFC,FG_WGS84,snap="near") #cropping to area of interest

projection(TFC)<-proj_Dab81 #projecting the raster

TFC<-setExtent(TFC,Extent_Dab81) #setting correct extent

writeRaster(TFC,filename=paste0(pathFD,"/Guinee_Dab81/Forest_Dab81/Tree_forest_area_Dab81.tif"),overwrite=T) #save raster

### CROPLAND ####

## Raster of percentage of cropland; resolution 1km

GDALinfo(".\\Guinee\\Cropland\\glc_shv10_02.tif") #to access metadata

Crops<-raster(".\\Guinee\\Cropland\\glc_shv10_02.tif") #load raster

Crops<-crop(Crops,FG_WGS84,snap="near") #cropping to area of interest

projection(Crops)<-proj_Dab81 #projecting the raster

Crops<-setExtent(Crops,Extent_Dab81) #setting correct extent

writeRaster(Crops,filename=paste0(pathFD,"/Guinee_Dab81/Cropland_Dab81/Cropland_Dab81.tif"),overwrite=T) #save raster

### CROPLAND : TREE COVER RATIO ####

## Using the raster of Forest and Cropland, estimate the ratio of cropland to forest cover

## The two rasters need to be loaded

Ratio<-overlay(Crops,TFC,fun=function (x,y){x/(x+y)})

projection(Ratio)<-proj_Dab81

writeRaster(Ratio,filename=paste0(pathFD,"/Guinee_Dab81/Forest_Crop_Ratio_Dab81/Forest_Crop_ratio_Dab81.tif"),overwrite=T)

### NDVI ####

## Files are MOD13A3 products from NASA. They are 4hdf files. Two tiles cover the area of interest

## See script MODIS_script which allows to opent a GUI to download MODIS images in batch

## See 'A_NDVI_script' for procedure to format hdf MODIS files to the final raster to use in MCE

### TREE COVER LOSS ####

## The raster is the proportion of forest cover lost between 2001 to 2012; resolution of 30m

## There are two tiles that cover the study area in Guinea.

## The two tiles were merged in ArcGis and the resulting raster is "ForestCoverLoss_13.tif"

## Crop to area of interest

FCL<-raster(paste0(wd,"/Guinee/TreeCoverLoss/ForestCoverLoss_13.tif")) #load raster

FCL<-projectRaster(FCL,crs=proj_WGS84) #to make sure it is in the correct projection

FCL_c<-crop(FCL,Extent_WGS84,snap="near") #cropping to area of interest

FC_loss<-setExtent(FC_loss,ext=Extent_WGS84)

FC_loss.p<-aggregate(FC_loss,fact=30, fun=mean) #aggregate to have resolution of 1km

FC_loss.p<-setExtent(FC_loss.p,ext=Extent_WGS84)

FC_loss.p<-resample(FC_loss.p,rast_WGS84,method="bilinear") # resampling is needed to get the exact resoltuion

FC_loss.p<-projectRaster(FC_loss,rast_Dab81) #reproject to Guinea area

writeRaster(FC_loss.p,filename=paste0(pathFD,"/Guinee_Dab81/ForestCoverLoss_Dab81/ForestCoverLoss2013_Dab81.tif"))

## However the resulting raster has 122 negative values (of 111069 total cells), because of the reprojection.

FC_loss.p[FC_loss.p<0] #see that negative values are very close to zero

FC_loss.B<-FC_loss

FC_loss.B[FC_loss.B < 0] <- 0 #reclassifying negative values as zeros

writeRaster(FC_loss.B,filename=paste0(pathFD,"/Guinee_Dab81/ForestCoverLoss_Dab81/ForestCoverLoss2013_Dab81_good.tif"))

### ROADS ####

# Roads, streets and path data are shapefiles downloaded from OpenStreetMap.

roads<-readOGR(dsn=paste0(wd,"/Guinee/Roads/Guinea_mainRoads"),layer="gin_trs_roads_osm")

roads<-spTransform(roads,proj_WGS84) #Reprojection

roads<-crop(roads,FG) #crop to extent area

writeOGR(roads,dsn=paste0(wd,"/Guinee/Roads/Guinea_mainRoads_crop"),layer="roads",driver="ESRI Shapefile") #save cropped shapefile

streets<-readOGR(dsn=paste0(wd,"/Roads/Guinea_street&paths"),layer="gin_trs_streets_osm")

streets<-spTransform(streets,proj_WGS84) #Reprojection

streets<-crop(streets,FG,snap=near)

writeOGR(streets,dsn=paste0(wd,"/Roads/Guinea_street&paths_crop"),layer="streets",driver="ESRI Shapefile")

#The above shapefiles were merged using ArcGis to have one shapefile with all roads and streets called "roadsANDstreet".

#Note that this are not in the correct projection for Guinea

### Projection to Dab81 projection to get the projected shapefile

roads<-readOGR(dsn=paste0(wd,"Guinee/Roads/roads&street"),layer="roadsANDstreet")

roads<-spTransform(roads,proj_Dab81)#Reprojection

writeOGR(roads,dsn=paste0(pathFD,"/Guinee_Dab81/Roads_Dab81/all_roads_Dab81"),layer="all_roads_Dab81",driver="ESRI Shapefile") #save reprojected shapefile

# The resulting shapefile above was used to estimate Euclidean distance in ArcGis using the raster "Tree_forest_area_Dab81.tif" to get resolution and extent.

# This raster has a relative difference of 0.000087 in relation to ymin;

# a mean relative difference of 0.0033 in relation to the number of rows and of 0.0015 in resolution.

# Therefore resampling was done to get exact extent and resolution as the other rasters

roads_81<-raster(paste0(pathFD,"/Guinee_Dab81/Roads_Dab81/Roads_raster_Dab81/Roads_EuclDist_Dab81.tif")) #load raster of Euclidean distance to roads

roads_81_res<-resample(roads_81,rast_Dab81,method="bilinear")

writeRaster(roads_81_res,paste0(pathFD,"/Roads_Dab81/Roads_raster_Dab81/roads_EuclDist_Dab81_goodRes.tif")) #save the raster of Euclidean distance in correct projection and resolution

### RIVERS ####

## The data is a shapefile of waterways in Guinea

rivers<-readOGR(dsn=paste0(wd,"/Guinee/Rivers/Guinea_WaterAreas"),layer="GIN_water_lines_dcw")

rivers_81<-spTransform(rivers,proj_Dab81) #Reprojection to Dab81

rivers_81<-crop(rivers_81,FG_Dab81,snap="near") #cropping to area of interest

writeOGR(rivers_81,dsn=paste0(pathFD,"/Guinee_Dab81/Rivers_Dab81/Rivers_Dab81"),layer="rivers_dab81",driver="ESRI Shapefile") #saving shapefile of rivers in Forested Guinea

# The resulting shapfile was used to calculate the Euclidean distance in ArcGis with the Tree_forest_area_Dab81.tif as raster to get resolution and extent.

# This raster has a relative difference of 0.000064 and 0.0011 in relation to ymax and ymin respectively;

# a mean relative difference of 0.0066 in relation to the number of rows and of 0.001 in resolution.

# Therefore resampling was done to get a raster with the exact extent and resolution as the others

rivers_81<-raster(paste0(pathFD,"/Guinee_Dab81/Rivers_Dab81/Rivers_raster/Rivers_EuclDist_Dab81.tif")) #load raster of Euclidean distance to rivers

rivers_81_res<-resample(rivers_81,rast_Dab81,method="bilinear")

writeRaster(rivers_81_res,paste0(pathFD,"/Rivers_Dab81/Rivers_raster/Rivers_EuclDist_Dab81_goodRes.tif")) #save the raster of Euclidean distance in correct projection and resolution

### PRECIPITATION ####

## Data is raster of montly rainfall in mm, resolution 0.05 degrees

## see Precipitation_script for a loop to process rasters

### TEMPERATURE ####

## Data is MODIS data of land surface temperature

## See Temperature_script to process temperature data and to estimate Temperature Annual Range and Annual Mean.

### POPULATION ####

## Data is raster of population density (ie. persons/pixel) in 2013 (for Guinea). Resolution of 100m.

Pop<-raster(paste0(wd,"/Guinee/Population/gin_ppp_2013.tif"))

Pop<-crop(Pop,Extent_WGS84) #cropping

Pop<-projectRaster(Pop,crs=proj_WGS84)

Pop_agg<-aggregate(Pop,fact=10, fun=mean) # aggregate to get resolution of 1km aprox

Pop_agg.res<-resample(Pop_agg,rast_WGS84,method="bilinear") #resampling to get exact resolution

Pop_81<-projectRaster(Pop_agg.res,rast_Dab81) #reproject to Guinea projection

writeRaster(Pop_81,paste0(pathFD,"Guinee_Dab81/Population_Dab81/Population2013_Dab81.tif")) #save raster

### VILLAGES ####

## Village for the study area in Guinea area data were obtained from OpenStreetMap with the tag = 'place', which are points

## Places were filtered to keep only villages (less than 10000 hab and more than a hamlet) and hamlet (100-200 hab, only with some places for housing and farms un rural areas)

## Thus there was a total of 2602 points which were rasterized and reclassified in ArcGis so that pixels where a village or hamlet is present has a value of 1 and 0 where there is none.

## The points and raster are already projected to the CRS Dabola 1981

village<-raster(paste0(wd,"Guinee/Villages/villages_raster/villagesEThamlet_raster.tif"))

village<-projectRaster(village,rast_Dab81)

writeRaster(village,filename=paste0(pathFD,"/Guinee_Dab81/Villages_Dab81/villages_notReclass.tif")) #save projected raster, but not reclassified

## The raster was reclassified ('villages_reclass.tif') so that villages =1 and all rest=No Data in ArcGis

## From the reclassified raster, the euclidean distance was calculated in ArcGis

dist_village<-raster(paste0(pathFD,"/Guinee_Dab81/Villages_Dab81/villages_EuclDist.tif")) #load raster of Euclidean distance from villages

dist_village<-projectRaster(dist_village,rast_Dab81) #to make sure that extent and resolution are the same as for other rasters

## The relationship with the risk of spillover of EBOV is the inverse, ie. it is assumed that the risk increases closer to the villages

## to calculate the inverse of the raster, the following was done:

dist_village_min<-cellStats(dist_village,'min') #get the min value

dist_village_max<-cellStats(dist_village,'max') #get the max value

dist_villageInv<-(1 - ((dist_village - dist_village_min) / (dist_village_max - dist_village_min))) * (dist_village_max - dist_village_min) + dist_village_min

writeRaster(dist_villageInv,filename=paste0(pathFD,"/Guinee_Dab81/Villages_Dab81/villages_EuclDistInv.tif"),overwrite=T) #save raster of the Inverse of the distance

### BUSHMEAT HUNTING AREAS ####

## It was assumed that hunting takes place preferrably in classed forests

## A map of classed forests was obtained from the report "Stratégie Nationale de gestion des éléphants en République de Guinée (2008)"

## This map was georeferenced and reclassified in ArcGis so that pixels in classed forests have a value of 1 and the rest has values of zero.

bush<-raster(paste0(wd,"Guinee/Bushmeat/classed_forests_dab81.tif")) #raster of classed forests

bush<-projectRaster(bush,rast_Dab81) #projected to Guinea projection

writeRaster(bush,filename=paste0(pathFD,"/Guinee_Dab81/Bushmeat_Dab81/Bushmeat_Hunting.tif"),overwrite=T) ## save raster

#The raster above was reclassified in ArcGis to make sure that all values > 0 are equal to 1, the resulting raster is called "BushmeatHunting_std"

bush<-raster(paste0(pathFD,"/Guinee_Dab81/Bushmeat_Dab81/BushmeatHunting_std.tif"))

bush<-projectRaster(bush,rast_Dab81) #make sure that projection is the correct one

writeRaster(bush,filename=paste0(pathFD,"/Guinee_Dab81/Bushmeat_Dab81/BushmeatHunting.tif"),overwrite=T) # save raster

#####################################################################################

### STANDARDIZE rasters for GUINEA so that all have values 0-1 ####

## All rasters need to be standardized (values between 0 and 1) to be able to be compared in MCE

## To standardize: X-min/max-min, where X is the raster, min and max are the minimum and maximum value in the raster, respectively

## A mask is used so that all cells outside the study area become NoData

pathFD<-paste0(pathFD,"/Guinee_Dab81/") #path to rasters already in the correct extent and projection for Guinea

# Eg.

FC_loss_81<-raster(paste0(pathFD,"/ForestCoverLoss_Dab81/ForestCoverLoss2013_Dab81.tif")) #load raster

FC_loss_81<-mask(FC_loss_81,mask=FG_Dab81) # mask

FC_loss_min<-cellStats(FC_loss_81,'min') # get min value of raster

FC_loss_max<-cellStats(FC_loss_81,'max') # get max value of raster

FC_loss_std<-(FC_loss_81-FC_loss_min)/(FC_loss_max-FC_loss_min) # standardize

writeRaster(FC_loss_std,paste0(pathFD,"/ForestCoverLoss_Dab81/ForestCoverLoss2013_std.tif"),overwrite=T) #save standardized raster

## The same procedure was applied to all rasters, so that they have values between 0 and 1.

###########################################################################################

#### FINAL LAYERS FOR ANALYSIS ####

## Rasters have been formatted from raw data and have been standardized (0 to 1)

## This script allows to check if all rasters have the same extent and resolution before applying the normalising functions.

wd="/.../" #path to working directory MTD

setwd(wd)

pathFD=paste0(wd,"/Formated_Data/") #path to folder with formatted rasters

## All layers must already have the same projection (eg.Dab81 for Guinea)

### Loading data in Dab81 Projection ####

load("AOI.Rdata")

## From 1_AOI_script, the following objects need te be created

Extent_Dab81

rast_Dab81

## Load rasters (e.g. with tree cover)

Tree_cover<-raster(paste0(pathFD,"Guinee_Dab81/Forest_Dab81/Tree_forest_area_std.tif"))

## To check that extent and resolution are the same for all rasters, each raster is compared with the initial Extent and raster in the Dabola 1981 projection.

## Here the raster for NDVI is checked

all.equal(extent(Extent_Dab81),extent(Tree_cover))

all.equal(ncol(rast_Dab81),ncol(Tree_cover))

all.equal(nrow(rast_Dab81),nrow(Tree_cover))

all.equal(res(rast_Dab81),res(Tree_cover))

###########################################################################################

### APPLYING FUNCTIONS TO STANDARDISED RASTERS ####

## This script is for applying the assumed function to each raster representing a risk factor.

## All rasters have been standardised (values 0 to 1)

## All rasters have the same resolution and extent

## These are the equations for each curve

## x in each function should be the corresponding raster

y1<- x

y2<- 1-x

y3 <- 1 - (2*x-1)^2

y4 <- (2*x-1)^2

y5 <- sqrt(1 - x^2)

sigm <- function(x) 1 / (1 + exp(-(10*(x-1/2))))

y6 <- (sigm(x) - sigm(0))/(sigm(1) - sigm(0))

y7 <- 1 - (sigm(x) - sigm(0))/(sigm(1) - sigm(0))

y8 <- (exp(5*x) - exp(0))/(exp(5) - exp(0))

y9 <- (exp(5*(1-x)) - exp(0))/(exp(5) - exp(0))

y10 <- sqrt(1 - (1-x)^2)

### To see the shape of each function run the following script ####

library(ggplot2)

library(ggpubr)

library(gridExtra)

x<-runif(500)

y1<- x

y2<- 1-x

y3 <- 1 - (2*x-1)^2

y4 <- (2*x-1)^2

y5 <- sqrt(1 - x^2)

sigm <- function(x) 1 / (1 + exp(-(10*(x-1/2))))

y6 <- (sigm(x) - sigm(0))/(sigm(1) - sigm(0))

y7 <- 1 - (sigm(x) - sigm(0))/(sigm(1) - sigm(0))

y8 <- (exp(5*x) - exp(0))/(exp(5) - exp(0))

y9 <- (exp(5*(1-x)) - exp(0))/(exp(5) - exp(0))

y10 <- sqrt(1 - (1-x)^2)

dat=data.frame(x,y1,y2,y3,y4,y5,y6,y7,y8,y9,y10)

par(mfrow=c(5,2))

p1<-ggplot(dat,aes(x=x, y=y1))+geom_line(color="blue", size=2)+theme_classic()+scale_x_continuous(name="",breaks=c(0,1))+

scale_y_continuous(name="",breaks=c(0,1))+theme(plot.title=element_text(hjust=0.5))+labs(title="1")

p2<-ggplot(dat,aes(x=x, y=y2))+geom_line(color="blue", size=2)+theme_classic()+scale_x_continuous(name="",breaks=c(0,1))+

scale_y_continuous(name="",breaks=c(0,1))+theme(plot.title=element_text(hjust=0.5))+labs(title="2")

p3<-ggplot(dat,aes(x=x, y=y3))+geom_line(color="blue", size=2)+theme_classic()+scale_x_continuous(name="",breaks=c(0,1))+

scale_y_continuous(name="",breaks=c(0,1))+theme(plot.title=element_text(hjust=0.5))+labs(title="3")

p4<-ggplot(dat,aes(x=x, y=y4))+geom_line(color="blue", size=2)+theme_classic()+scale_x_continuous(name="",breaks=c(0,1))+

scale_y_continuous(name="",breaks=c(0,1))+theme(plot.title=element_text(hjust=0.5))+labs(title="4")

p5<-ggplot(dat,aes(x=x, y=y7))+geom_line(color="blue", size=2)+theme_classic()+scale_x_continuous(name="",breaks=c(0,1))+

scale_y_continuous(name="",breaks=c(0,1))+theme(plot.title=element_text(hjust=0.5))+labs(title="5")

p6<-ggplot(dat,aes(x=x, y=y6))+geom_line(color="blue", size=2)+theme_classic()+scale_x_continuous(name="",breaks=c(0,1))+

scale_y_continuous(name="",breaks=c(0,1))+theme(plot.title=element_text(hjust=0.5))+labs(title="6")

p7<-ggplot(dat,aes(x=x, y=y5))+geom_line(color="blue", size=2)+theme_classic()+scale_x_continuous(name="",breaks=c(0,1))+

scale_y_continuous(name="",breaks=c(0,1))+theme(plot.title=element_text(hjust=0.5))+labs(title="7")

p8<-ggplot(dat,aes(x=x, y=y10))+geom_line(color="blue", size=2)+theme_classic()+scale_x_continuous(name="",breaks=c(0,1))+

scale_y_continuous(name="",breaks=c(0,1))+theme(plot.title=element_text(hjust=0.5))+labs(title="8")

p9<-ggplot(dat,aes(x=x, y=y9))+geom_line(color="blue", size=2)+theme_classic()+scale_x_continuous(name="",breaks=c(0,1))+

scale_y_continuous(name="",breaks=c(0,1))+theme(plot.title=element_text(hjust=0.5))+labs(title="9")

p10<-ggplot(dat,aes(x=x, y=y8))+geom_line(color="blue", size=2)+theme_classic()+scale_x_continuous(name="",breaks=c(0,1))+

scale_y_continuous(name="",breaks=c(0,1))+theme(plot.title=element_text(hjust=0.5))+labs(title="10")

par(xpd=NA)

figure<-grid.arrange(p1,p2,p3,p4,p5,p6,p7,p8,p9,p10,nrow=5)

annotate_figure(figure,

bottom=text_grob("Risk factor", face="bold",size=14),

left=text_grob("Posibility of EBOV spillover",face="bold",size=14,rot=90))

## Applying the functions:

library(raster)

library(rgdal)

library(sp)

library(rgeos)

library(gdalUtils)

wd="/.../" #path to working directory MTD

setwd(wd)

pathFD=paste0(wd,"/Formated_Data/") #path to folder with formatted rasters

path_data<-paste0(pathFD,"Guinee_Dab81/") # path for rasters of study area (eg. Guinea)

path_Out<-paste0(wd,"/Rasters_MCE/")

### Environmental risk factors ####

### Forest Cover####

pathOut_Env<-paste0(path_Out,"/Guinee/Environment") #path to output rasters of environmental variables for Guinee

## function: y3 <- 1-(2*x-1)^2

Tree_cover<-raster(paste0(path_data,"/Forest_Dab81/Tree_forest_area_std.tif")) #load raster Guinee

x<-Tree_cover

Tree_cover <- 1-(2*x-1)^2 #apply function

writeRaster(Tree_cover,paste0(pathOut_Env,"/Tree_cover.tif"),overwrite=T)

### NDVI ###

## function: y3 <- 1-(2*x-1)^2

## It has been already applied, see NDVI_script

### Cropland ####

## function: y3 <- 1-(2*x-1)^2

Crops<-raster(paste0(path_data,"/Cropland_Dab81/Cropland_std.tif")) #load raster Guinee

x<-Crops

Crops<-1-(2*x-1)^2

writeRaster(Crops,paste0(pathOut_Env,"/Crops.tif"),overwrite=T)

### Ratio Cropland: forest cover ####

## function: y3 <- 1-(2*x-1)^2

Ratio<-raster(paste0(path_data,"/Forest_Crop_ratio_Dab81/ForestCropRatio_std.tif")) #load raster

x<-Ratio

Ratio<-1-(2*x-1)^2

writeRaster(Ratio,paste0(pathOut_Env,"/CropForestRatio.tif"),overwrite=T)

### Loss of forest cover ####

## function: y3 <- 1-(2*x-1)^2

Forest_loss<-raster(paste0(path_data,"/ForestCoverLoss_Dab81/ForestCoverLoss2013_std.tif"))

x<-Forest_loss

Forest_loss<-1-(2*x-1)^2

writeRaster(Forest_loss,paste0(pathOut_Env,"/Forest_loss.tif"),overwrite=T)

### Distance to rivers ####

## function: y5 <- sqrt(1 - x^2)

rivers<-raster(paste0(path_data,"/Rivers_Dab81/Rivers_raster/Rivers_EuclDist_std.tif"))

x<-rivers

rivers<-sqrt(1 - x^2)

writeRaster(rivers,paste0(pathOut_Env,"/Rivers.tif"),overwrite=T)

### Distance to roads ####

## function: y2<- 1-x

roads<-raster(paste0(path_data,"/Roads_Dab81/roads_raster_Dab81/roads_EuclDist_std.tif"))

x<-roads

roads<-1-x

writeRaster(roads,paste0(pathOut_Env,"/Roads.tif"),overwrite=T)

### Human population density ####

## function: y3 <- 1-(2*x-1)^2

Pop<-raster(paste0(path_data,"/Population_Dab81/Population2013_std.tif"))

x<-Pop

Pop<- 1-(2*x-1)^2

writeRaster(Pop,paste0(pathOut_Env,"/PopulationContactWildlife.tif"),overwrite=T)

### Climatic factors ####

pathOut_Climate<-paste0(path_Out,"/Guinee/Climate/") #path to output rasters of climate variables for Guinee

### Precipitation ####

## See Precipitation_script used to process all rainfall rasters in one go, including the step of applying the function below

## function: y6 <- (sigm(x) - sigm(0))/(sigm(1) - sigm(0))

### Annual Temperature Range ####

## function: y7 <- 1-(sigm(x)-sigm(0))/(sigm(1)-sigm(0))

TempRange<-raster(paste0(path_data,"/Temperature_Dab81/AnnualTempRange_2013_std.tif"))

x<-TempRange

TempRange<- 1-(sigm(x)-sigm(0))/(sigm(1) - sigm(0))

writeRaster(TempRange,paste0(pathOut_Climate,"TempRange2013.tif"),overwrite=T) #change file name according to place and year

### Mean Annual Temperature ####

## function: y1<- x (no need to apply function to raster, since it is already standardized and the function is increasing linear)

Temp<-raster(paste0(path_data,"/Temperature_Dab81/MeanTemp_2013_std.tif"))

writeRaster(Temp,paste0(pathOut_Climate,"MeanTemp2003.tif"),overwrite=T) #change file name according to place and year

### Risk factors associated with bushmeat###

pathOut_Bushmeat<-paste0(path_Out,"/Guinee/Bushmeat/") #path to output rasters of climate variables for Guinee

### Distance to villages ####

## Distance to village is used as a proxy for the bushmeat trade/consumption and for the presence of the domestic animals. However the relationship between it and spillover of EBOV is not considered the same.

dist_village<-raster(paste0(path_data,"/Villages_Dab81/villagesEuclDistInv_std.tif")) #raster of Euclidean distance from villages in Guinee

## For bushmeat trade/ consumption the function is y1<- x

bushmeat_trade<-dist_village #(no need to apply function to raster, since it is already standardized and the function is increasing linear)

writeRaster(bushmeat_trade,paste0(pathOut_Bushmeat,"BushmeatTrade.tif"),overwrite=T)

## For the presence of domestic animals the function is y10 <- sqrt(1-(1-x)^2)

domestic_animals<-dist_village

x<-domestic_animals

domestic_animals<-sqrt(1-(1-x)^2)

writeRaster(domestic_animals,paste0(pathOut_Bushmeat,"Domestic_animals.tif"),overwrite=T)

### Bushmeat hunting ####

##function: y1<- x

bushmeat_hunting<-raster(paste0(path_data,"/Bushmeat_Dab81/BushmeatHunting_std.tif"))#(no need to apply function to raster, since it is already standardized and the function is increasing linear)

writeRaster(bushmeat_hunting,paste0(pathOut_Bushmeat,"BushmeatHunting.tif"),overwrite=T)

### Human population density - bushmeat ####

## the relationship as a risk factor associated to bushmeat is y1<- x

Pop<-raster(paste0(path_data,"/Population_Dab81/Population2013_std.tif")) #no need to apply function as raster is already standardized and the function is increasing linear

writeRaster(Pop,paste0(pathOut_Bushmeat,"Population_bushmeat.tif"),overwrite=T)

###########################################################################################

### APPLY MULTICRITERIA EVALUATION ###

## First four maps are created:

# species map (contact with species associated with EBOV spillover)

# environmental risk factors map

# climatic risk factors map

# bushmeat risk factors map

## Weight for each risk factors is needed (from survey or literature review)

wd="/.../" #path to working directory

setwd(wd)

### E.g. Suitability maps for Guinea ####

## Paths to save results

path_Results<-paste0(wd,"/Results/")

path_ResGuinee<-paste0(path_Results,"/ResultsGuinee/") #path to save resulting maps

## Paths to load rasters

path_MCE<-paste0(wd,"/Donnees/Rasters_MCE/Guinee/")

path_species<-paste0(path_MCE,"Species/")

path_climate<-paste0(path_MCE,"Climate/")

path_environment<-paste0(path_MCE,"Environment/")

path_bushmeat<-paste0(path_MCE,"Bushmeat/")

### Species suitability map ####

species<-list.files(paste0(path_species)) #list of group of species

sp.weights<-c(0.125,0.375,0.375,0.125) #Weights for each species group. This needs to be in the order of the rasters (see command list.files above)

species_stack<-stack(paste0(path_species,species))

species_multip<-sp.weights*species_stack #multiplying each raster with its corresponding weight

species_map<-overlay(species_multip, fun=sum, unstack=T,

filename=paste0(path_ResGuinee,"species_map.tif"),overwrite=T)

### Environmental suitability map ####

environment<-list.files(paste0(path_environment))

env.weights<-c(0.096,0.048,0.255,0.096,0.167,0.032,0.05,0.255) #weight for each environmental risk factor. Needs to be in the order of the rasters

env_stack<-stack(paste0(path_environment,environment))

env_multip<-env.weights*env_stack

environmet_map<-overlay(env_multip,fun=sum,unstack=T,

filename=paste0(path_ResGuinee,"/environment_map_dec2013.tif"),overwrite=T)

## For the time series, the only raster that changes each month is the NDVI for the environmental map.

### Environmental suitability map ####

climate<-list.files(paste0(path_climate))

climate.weights<-c(0.686,0.102,0.211)

climate_stack<-stack(paste0(path_climate,climate))

climate_multip<-climate.weights*climate_stack

climate_map<-overlay(climate_multip,fun=sum,unstack=T,

filename=paste0(path_ResGuinee,"/climate_map_dec2013.tif"),overwrite=T) #change name according to month used for rainfall

## For the time series, the only raster that changes each month is the rainfall

### Bushmeat suitability map ####

bushmeat<-list.files(paste0(path_bushmeat))

bushmeat.weights<-c(0.38,0.38,0.062,0.179)

bushmeat_stack<-stack(paste0(path_bushmeat,bushmeat))

bushmeat_multip<-bushmeat.weights*bushmeat_stack

bushmeat_map<-overlay(bushmeat_multip,fun=sum,unstack=T,

filename=paste0(path_ResGuinee,"bushmeat_map.tif"),overwrite=T)

### EBOV Suitability Map ####

## Make sure that a map for species, climate, environment and bushmeat are in the same folder (eg. 'MapsRunMCE').

## Different species, environment, climate or bushmeat maps can be used to build different suitability maps.

path_maps<-"/...MapsRunMCE/"

maps<-list.files(paste0(path_maps))

maps_stack<-stack(paste0(path_maps,maps))

EBOV_spillover<-mean(maps_stack)

writeRaster(EBOV_spillover,paste0(path_ResGuinee,"/FinalMaps/Guinee_Spillover_dec2013.tif"),overwrite=T)

#####################################################################################

### NDVI Script ###

## Script to process hdf files for NDVI

## files are product MOD13A3, resolution 1km

## there are two tiles that cover the area of interest in Guinea

## First hdf files are converted to geotiff and then they are merged into one GeoTiff file

## Then standardizing and normalising functions are applied

library(raster)

library(gdalUtils)

wd="/.../" #path to working directory

setwd(wd)

pathMod=paste0(wd,"/.../MOD13A3_2013/") #path to hdf files from 2013

dir.create(paste0(wd,"/.../NDVI_2013_geotiff/")) #create folder to save tif files

pathGeo=paste0(wd,"/.../NDVI_2013_geotiff/") #path to save rasters in the folder created above

dir.create(paste0(wd,"/.../NDVI_full_2013/")) #create folder to save tif files

pathMos=paste0(wd,"/.../NDVI_full_2013/") #path to save mosaic of both tiles

# Convert the two tiles into Geotiff

# Note that the name of each hdf file needs to be changed accordingly

gdalinfo(paste0(pathMod,"/2013.01/MOD13A3.A2013001.h16v08.006.2015254151802.hdf")) #get data on the file

## Processing file for December 2013

sds<- get_subdatasets(paste0(pathMod,"2013.12/MOD13A3.A2013335.h16v08.006.2015272025719.hdf")) #get layers (subdatasets) of file

gdal_translate(sds[1],dst_dataset=paste0(pathGeo,"/NDVI_dec2013_h16v08.tif")) #to convert the file in a GeoTiff. The first layer (ie. sds[1]) contains the raster of NDVI

sds<- get_subdatasets(paste0(pathMod,"2013.12/MOD13A3.A2013335.h17v08.006.2015272024451.hdf")) #repeat process for 2nd tile

gdal_translate(sds[1],dst_dataset=paste0(pathGeo,"/NDVI_dec2013_h17v08.tif"))

## Using mosaic_rasters() to merge the two tiles into one

r1<-paste0(pathGeo,"NDVI_dec2013_h16v08.tif")

r2<-paste0(pathGeo,"NDVI_dec2013_h17v08.tif")

mosaic_rasters(c(r1,r2),dst_dataset=paste0(pathMos,"/NDVI_Dec2013.tif"))

## This procedure needs to be repeated for the NDVI for each month in 2013.

## But see script MODIS_script which allows to opent a GUI to download MODIS images in batch, merge several tiles and save them as GeoTiff

### Standardise and apply function to NDVI rasters ####

## Once all hdf files have been transformed into GeoTiff and merged, they are standardized and normalized

## from 1.AOI_script the following objects are created:

load("AOI.RData")

proj_Dab81

FG_Dab81

Extent_Dab81

rast_Dab81

dir.create(paste0(wd,"/.../NDVI_Dab81")) #create folder to save formatted rasters

pathSave=paste0(wd,"/.../NDVI_Dab81/") #path to folder to save formatted rasters

pathMos=paste0(wd,"/.../NDVI_full_2013/") #path to get NDVI rasters already merged

list_NDVI=list.files(pathMos)

for (i in 1:length(list_NDVI)){

NDVI<-list_NDVI[i]

NDVI_rast<-raster(paste0(pathMos,NDVI)) #loading raster

NDVI_rast<-NDVI_rast*0.0001 #to get values of NDVI between -1 to 1 (see MODIS documentation for product MOD13A3)

NDVI_rast<-projectRaster(NDVI_rast,crs=proj_Dab81) #reproject raster

NDVI_rast<-crop(NDVI_rast,FG_Dab81)

NDVI_rast<-setExtent(NDVI_rast,ext=Extent_Dab81) #setting correct extent

NDVI_rast<-resample(NDVI_rast,rast_Dab81,method="bilinear") # resampling to get good resolution

NDVI_rast<-mask(NDVI_rast,mask=FG_Dab81)

NDVI_min<-cellStats(NDVI_rast,'min')

NDVI_max<-cellStats(NDVI_rast,'max')

NDVI_std<-(NDVI_rast-NDVI_min)/(NDVI_max-NDVI_min) # standardize (0 to 1)

x<-NDVI_std

NDVI_rast<-1-(2*x-1)^2 #normalising function

writeRaster(NDVI_rast,paste0(pathSave,substr(NDVI,1,nchar(NDVI)-4),"_std.tif"),overwrite=T)

}

## To check that rasters are the same resolution as all the others

all.equal(extent(Extent_Dab81),extent(NDVI))

all.equal(ncol(rast_Dab81),ncol(NDVI))

all.equal(nrow(rast_Dab81),nrow(NDVI))

all.equal(res(rast_Dab81),res(NDVI))

###########################################################################################

#### Precipitation script ###

## Data is monthly rainfall in mm; resolution 0.05 degrees

## Script to find the driest and wettest month

## Then the rasters are standardized to be used in MCE

wd="/.../" #path to working directory

setwd(wd)

## From 1_AOI_script create the Extent and rasters of the area of interest for reprojection and cropping

load (paste0(wd,"/Donnees/AOI.RData"))

Extent_WGS84

rast_WGS84

rast_Dab81

## Rainfall for Guinee forestiere 2013 ####

wd="/.../" #path to working directory

setwd(wd)

## function: y6 <- (sigm(x) - sigm(0))/(sigm(1) - sigm(0)) # function to standardize (ie. assumed relationship between Ebola spillover and rainfall)

sigm <- function(x) 1 / (1 + exp(-(10*(x-1/2)))) #to use in function

dir.create(paste0(wd,"/.../Rainfall")) #create folder output rasters

path_rainfall<-paste0(wd,"/.../Rainfall/") #path for output rasters

rain_list=list.files(paste0(wd,"Donnees/Guinee/Precipitation/CHIRPS/Chirps_2013"))

for (i in 1:length(rain_list)){

rain=rain_list[i]

rain_rast<-raster(paste0(wd,"/Donnees/Guinee/Precipitation/CHIRPS/Chirps_2013/",rain))

rain_rast<-crop(rain_rast,Extent_WGS84) #cropping

rain_rast<-disaggregate(rain_rast,fact=5,method="bilinear") # disaggregate to a resolution of 1km aprox

rain_rast<-resample(rain_rast,rast_WGS84,method="bilinear") # resampling to get the correct resolution

rain_rast<-projectRaster(rain_rast,rast_Dab81) # Reprojection for Dab81 for Guinea

rain_rast<-mask(rain_rast,mask=FG_Dab81) # mask raster

rain_min<-cellStats(rain_rast,'min') # calculate min for raster

rain_max<-cellStats(rain_rast,'max') # calculate max for raster

rain_std<-(rain_rast-rain_min)/(rain_max-rain_min) #standardize between 0 and 1

x<-rain_std

rain_func<-(sigm(x) - sigm(0))/(sigm(1) - sigm(0)) # apply sigmoid function

writeRaster(rain_func,paste0(path_rainfall,substr(rain,1,7),substr(rain,13,19),".tif"),overwrite=T) #save raster

}

###########################################################################################

### Temperature script ####

## Temperatures from Modis MOD11A2 product (8 days surface temperature, 1km resolution)

## Temperature needs to be multiplied by a factor of 0.02 to be in Kelvin degrees

library(raster)

library(rgdal)

library(sp)

library(rgeos)

library(gdalUtils)

wd="/.../" #path to working directory

setwd(wd)

path_Temperature=paste0(wd,"/Temperature/")

path_FG=paste0(wd,"/.../") #path to folder to save rasters

sr<-"+init=epsg:3462 +proj=utm +zone=29 +a=6378249.2 +b=6356515 +towgs84=-83,37,124,0,0,0,0 +units=m +no_defs" ##projection Guinea

## Load objects from AOI script ##

load("AOI.RData")

FG_Dab81 #from AOI script

### Min and Max annual temperature ####

## Loop to reproject, crop and convert to Celsius and save in a list of rasters

path_temp<-paste0(path_Temperature) #path to raster files

list_temp=list.files(path_temp)

raster_temp=as.list(list_temp[1:46]) #list to put the output rasters of the loop (will depend on number of output rasters)

for(i in 1:length(list_temp)){

Temp=list_temp[i]

raster_temp[[i]]<-raster(paste0(path_temp,Temp)) #loading rasters

raster_temp[[i]]<- projectRaster(raster_temp[[i]], crs = sr) #Project Raster

raster_temp[[i]]<-crop(raster_temp[[i]],FG_Dab81) #crop raster

raster_temp[[i]]<-raster_temp[[i]]*0.02 #multiply by factor (see MODIS documentation)

raster_temp[[i]]<-raster_temp[[i]]-273.15 #convert to Celsius

}

## Use the list of files above to find max and min temperatures

TempStack_Guinee<-stack(raster_temp) #creating stack of all temperatures within a year

MaxTemp_Guinee<-calc(TempStack_Guinee,fun=max)

MinTemp_Guinee<-calc(TempStack_Guinee,fun=min)

TempRange_Guinee<-MaxTemp_Guinee-MinTemp_Guinee #calculate temperature range

TempRange_Guinee<-projectRaster(TempRange_Guinee,rast_Dab81,method="bilinear") #resampling and putting raster into the right projection

writeRaster(TempRange_Guinee,paste0(path_FG,"/AnnualTempRange_2013.tif"),overwrite=T)

## Use the list of files above to find mean temperature removing NA values

TempStack_Guinee<-stack(raster_temp) #creating stack of temperatures within a year

MeanTemp<-mean(TempStack_Guinee, na.rm=T) #calculate mean temperature

MeanTemp<-projectRaster(MeanTemp,rast_Dab81, method="bilinear") #putting raster in same extent and resolution as the others

writeRaster(MeanTemp,filename=paste0(path_FG,"/MeanTemp_2013.tif"),overwrite=T)

### Standardize rasters ####

# TempRange<-raster(paste0(path_FG,"/Temperature_Dab81/AnnualTempRange_2013.tif"))

TempRange<-TempRange_Guinee

TempRange<-mask(TempRange,mask=FG_Dab81)

TempRange_min<-cellStats(TempRange,'min')

TempRange_max<-cellStats(TempRange,'max')

TempRange_std<-(TempRange-TempRange_min)/(TempRange_max-TempRange_min)

writeRaster(TempRange_std,paste0(path_FG,"/Temperature_Dab81/AnnualTempRange2013_std.tif"),overwrite=T)

MeanTemp<-raster(paste0(path_FG,"/Temperature_Dab81/MeanTemp_2013.tif"))

MeanTemp<-mask(MeanTemp,mask=FG_Dab81)

MeanTemp_min<-cellStats(MeanTemp,'min')

MeanTemp_max<-cellStats(MeanTemp,'max')

MeanTemp_std<-(MeanTemp-MeanTemp_min)/(MeanTemp_max-MeanTemp_min)

writeRaster(MeanTemp_std,paste0(path_FG,"/Temperature_Dab81/MeanTemp2013_std.tif"),overwrite=T)

### Apply normalizing functions

pathOut_Climate<-paste0(path_FG,"/Rasters_MCE/")

## function: y7 <- 1-(sigm(x)-sigm(0))/(sigm(1)-sigm(0))

sigm <- function(x) 1 / (1 + exp(-(10*(x-1/2))))

# TempRange_std<-raster(paste0(path_FG,"/Temperature_Dab81/AnnualTempRange2013_std.tif")) #load raster

x<-TempRange_std

TempRange_stdF<- 1-(sigm(x)-sigm(0))/(sigm(1) - sigm(0))

writeRaster(TempRange_stdF,paste0(pathOut_Climate,"/Temperature/TempRange2013.tif"),overwrite=T)

### MEAN ANNUAL TEMPERATURE ####

## function: y1<- x (no need to apply function to raster, since it is already standardized and the function is increasing linear)

# Temp<-raster(paste0(path_data,"/Temperature_Dab81/MeanTemp2013_std.tif")) #load raster

writeRaster(MeanTemp_std,paste0(pathOut_Climate,"/Temperature/MeanTemp2013.tif"))

###########################################################################################

### Species distribution maps ####

# Distribution maps (rasters of presence/absence) are loaded

# Each species map is multiplied by the average probability of it being a host or implicated in spillover according to experts or decided upon a given criteria.

# Eg. For the Franquet's epauletted fruit bat (Epomops franqueti) the average probability given was 8.82.

# Thus in the original map where presence = 1, after multiplication, those pixels will be 8.82.

# Absence is zero, so it will remain zero after multiplication

# Here we use weights decided from criteria and information found in a literature review.

library(raster)

wd="/.../" #path to working directory

setwd(wd)

load(paste0(wd,"/Donnees/AOI.RData"))

#Objects from AOI_script

proj_Dab81 #projection for Guinea

FG_Dab81 #shape of study area in Guinea

pathFD=paste0(wd,("/Donnees/Formated_Data/Guinee_Dab81/"))

dir.create(paste0(wd,"/Results/ResultsGuinee/SpeciesWeights/")) #create folder to save rasters of species multiplied by their weights

dir.create(paste0(wd,"/Results/ResultsGuinee/SpeciesWeights/Multiplied_Sp/")) #create folder to save rasters of species multiplied by their weights

dir.create(paste0(wd,"/Results/ResultsGuinee/SpeciesWeights/FinalRasters_Sp/")) #create folder to save final rasters of species

path_Out=paste0(wd,"/Results/ResultsGuinee/SpeciesWeights/Multiplied_Sp/") #path to save multiplied rasters in the folder just created

path_OutFinal=paste0(wd,"/Results/ResultsGuinee/SpeciesWeights/FinalRasters_Sp/") #path to save multiplied rasters in the folder just created

### Fruit bats ####

## Average probability for fruit bat species

# African straw-coloured fruit bat (Eidolon helvum) 3

# Gambian epauletted fruit bat (Epomophorus gambianus) 3

# Ethiopian epauletted fruit bat (Epomophorus labiatus) 3 #not in Guinee forestiere

# Wahlberg's epauletted fruit bat (Epomophorus wahlbergi) 3 #not in Guinee forestiere

# Franquet's epauletted fruit bat (Epomops franqueti) 5 #not in Guinee forestiere

# Hammer-headed frut bat (Hypsignathus monstrosus) 5

# Angolan fruit bat (Lissonycteris angolensis) 3

# Peter's dwarf epauletted fruit bat (Micropteropus pusillus) 3

# Little collared fruit bat (Myonycteris torquata) 5 #not in Guinee forestiere

# Egyptian fruit bat (Rousettus aegyptiacus) 3

dir.create(paste0(wd,"/Results/ResultsGuinee/SpeciesWeights/Multiplied_Sp/Frugivorous_bats_mult")) #create folder to save multiplied raster for this group

pathSp=paste0(pathFD,"/Species_Dab81/Frugivorous_bats_Dab81/") #path to access data of each group of species

list_Species<-list.files(pathSp) #list of species in each group

Avg_host<-c(3,3,3,3,5,5,3,3,5,3) #Average probability. This has to be in the same order as R will read the tif files. See list_Species to see order

for (i in 1:length(list_Species)){

Species=list_Species[i] #get species

Raster_Sp<-raster(paste0(pathSp,Species)) #load raster of species

Species_mult<-Raster_Sp*Avg_host[i] #multiply by average probability of being host

Species_mult<-Species_mult*0.1 #multiply by 0.1 to get values between 0 and 1

Species_mult=mask(Species_mult,mask=FG_Dab81) #mask

writeRaster(Species_mult,filename=paste0(path_Out,"/Frugivorous_bats_mult/",substr(Species,1,nchar(Species)-4),"_mult.tif"),overwrite=T) #save raster

}

# Remove species not present in the study area and calculate final raster for the group

Sp_not_AOI<-c("Epomophorus_labiatus_Dab81_mult.tif","Epomophorus_wahlbergi_Dab81_mult.tif","Epomops_franqueti_Dab81_mult.tif","Myonycteris_torquata_Dab81_mult.tif") #species not present in the area of interest

Avg.Species=list.files(paste0(path_Out,"Frugivorous_bats_mult/")) #list of average probability rasters of species

Avg.Species=Avg.Species[!Avg.Species %in% Sp_not_AOI] #removing spp that are not in the AOI.

stack_Avg.Species<-stack(paste0(path_Out,"Frugivorous_bats_mult/",Avg.Species)) #stack raster of all species within a group that are in AOI

sum_frugi_bats<-sum(stack_Avg.Species) #adding rasters within the stack

final_frug<-sum_frugi_bats/length(Avg.Species) # dividing by the total of species within the group that are in AOI

writeRaster(final_frug,filename=paste0(path_OutFinal,"/Frugivorous_bats_final.tif"),overwrite=T) #save raster

#### REPEAT PROCEDURE FOR THE OTHER GROUPS ####

### Standardize raster of species distribution ####

pathSpRast=path_OutFinal #path to rasters of spp already multiplied by the weights for each species

list_Species<-list.files(pathSpRast) #list of species rasters

for (i in 1:length(list_Species)){

Species=list_Species[i]

Raster_Sp<-raster(paste0(pathSpRast,Species)) #load rasters

Sp_min<-cellStats(Raster_Sp,'min') #get min

Sp_max<-cellStats(Raster_Sp,'max') #get max

Sp_std<-(Raster_Sp-Sp_min)/(Sp_max-Sp_min) #standardise (0-1)

writeRaster(Sp_std,filename=paste0(pathSpRast,substr(Species,1,nchar(Species)-4),"_std.tif")) #save raster

###########################################################################################

#### SENSITIVITY ANALYSIS by category of risk factor ####

## Analysis by category of risk factor to produce the uncertainty surface

## Previously, suitability maps need to be produced by increasing/decreasing the weight of the category of factors.

## An index of absolute delta is calculated as delta =abs(pixel value of final map - pixel value of weight variation map)/pixel value of final map

## The final map refers to the final spillover suitability map; the weight variation map refers to the output map when the weight of each category of risk factor was increased or decreased

## This is done only for a variation of 25% in weight.

## Since there are four types of factors (environment, climate, bushmeat and species), 8 maps are produced in the end

## The map of the mean and the map of the SD of these maps are created.

library(raster)

wd="/.../" #path to working directory

setwd(wd)

pathSA<-paste0(wd,"/Results_maps/Guinee/SensitivityAnalysis/") #path MTD

path_byT<-paste0(pathSA,"/SA_byType/")

## Loop to calculate the delta maps for the 8 maps where weight was changed by 25%

path_maps<-paste0(path_byT,"/Maps_tiff/") #path to maps when weights were increased/decreased

list_maps<-list.files(paste0(path_maps)) #list of maps

list_out<-list() #list for output maps

Suitmap<-raster(paste0(path_byT,"/Maps_tiff/Suit_Spillover.tif")) #Final suitability map

## Estimate delta

for (i in 1:length(list_maps)){

map=list_maps[i]

R1=Suitmap

R2=raster(paste0(path_maps,map))

delta=overlay(R1,R2,fun=function(r1,r2){return(abs(r1-r2)/r1)}) # calculate delta

list_out[[i]]=delta

names(list_out)[i]= paste0("delta_",map) #name output map

}

SAMaps_stack<-stack(list_out)

MeanMap<-calc(SAMaps_stack,fun=mean,filename=paste0(path_byT,"/Mean_WV_25.tif")) #calculate the Mean map

SDMap<-calc(SAMaps_stack,fun=sd,filename=paste0(path_byT,"/StDev_WV_25.tif")) #calculate the SD map

### END ###

#### SENSITIVITY ANALYSIS by risk factor within each category ####

## This is done by varying the weight of each risk factor within its corresponding category.

## Here it is done for the Environmental Risk factors. The same procedure is followed for the other three categories (i.e. Climate, Bushmeat, Species)

## Previously suitability maps where the weight of each risk factors is increased/decreased by 25% were created

## An index of absolute delta is calculated as delta =abs(pixel value of final map - pixel value of weight variation map)/pixel value of final map

## The final map refers to the final environmental suitability map; the weight variation map refers to the output map when the weight of each factor is increased or decreased

## Mean and SD are calculated for the delta values of each increase/decrease in weight of 25%

library(raster)

wd="/.../" #path to working directory

setwd(wd)

pathSA<-paste0(wd,"/.../SensitivityAnalysis/") #

path_Env<-paste0(wd,"/Environment/") # path to maps of environmental risk factors

ENVmap<-raster(paste0(path_Env,"/Maps_Env_tiff/Suit_EnvironmentMap.tif")) #Final Environmental suitability map

path_RF<-paste0(path_Env,"/Maps_Env_tiff/CropForestRatio/") #path to risk factor

list_maps<-list.files(paste0(path_RF))

Mean_list<-list() #list to save Mean values

SD_list<-list() #list to save SD values

for (i in 1:5){

map_D=list_maps[i]

map_I=list_maps[i+5]

r1=ENVmap

r_D=raster(paste0(path_RF,map_D))

r_I=raster(paste0(path_RF,map_I))

delta_D=overlay(r1,r_D,fun=function(r1,r2){return(abs(r1-r2)/r1)}) #calculate delta of decrease in weight

delta_I=overlay(r1,r_I,fun=function(r1,r2){return(abs(r1-r2)/r1)}) #calculate delta of increase in weight

vD=as.vector(delta_D)

vI=as.vector(delta_I)

vf=c(vD,vI)

Mean_list[[map_I]]=mean(vf,na.rm=T)

SD_list[[map_I]]=sd(vf,na.rm=T)

names(Mean_list)[i]<-paste0(map_I,"_Mean")

names(SD_list)[i]<-paste0(map_I,"_SD")

}

Stats<-do.call(rbind.data.frame,Map('c',Mean_list, SD_list))

names(Stats)<-c("Mean","SD")

Stats$WV<-paste0(rep("CFR_",5),seq(05,25,5))

write.csv(Stats,paste0(path_Env,"/Abs_Delta_Stats/CropForestRatio.csv"),row.names=F)

### The same procedure is used for each risk factor
